# Supplementary figures and images for: Learning the rules of collective cell migration using deep attention networks
Source: PLoS Comput Biol. 2022 Apr 27;18(4):e1009293. doi: 10.1371/journal.pcbi.1009293 (PMC9106212; doi:10.1371/journal.pcbi.1009293)

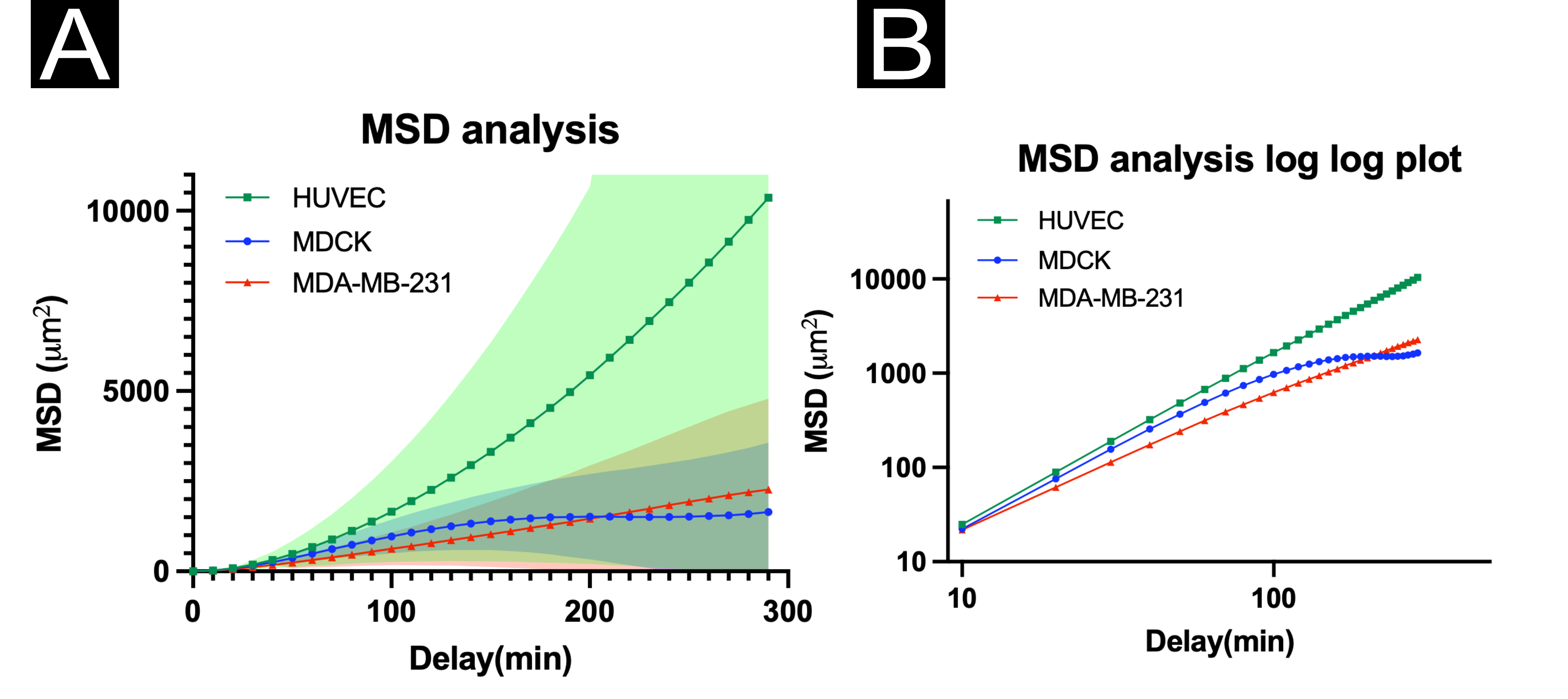

Supplement: S1 Fig — Mean squared displacement (MSD) over time. (A) Linear-scale MSD to emphasize distinct differences in MSD trajectories; shaded zones indicate the weighted standard deviation of the individual MSD trajectories (see MSDAnalyzer software). (B) Log-scale of MSD for a more traditional rendering of the MSD that highlights the long-lag caged behavior of MDCKs. (JPG) [file pcbi.1009293.s001.jpg]

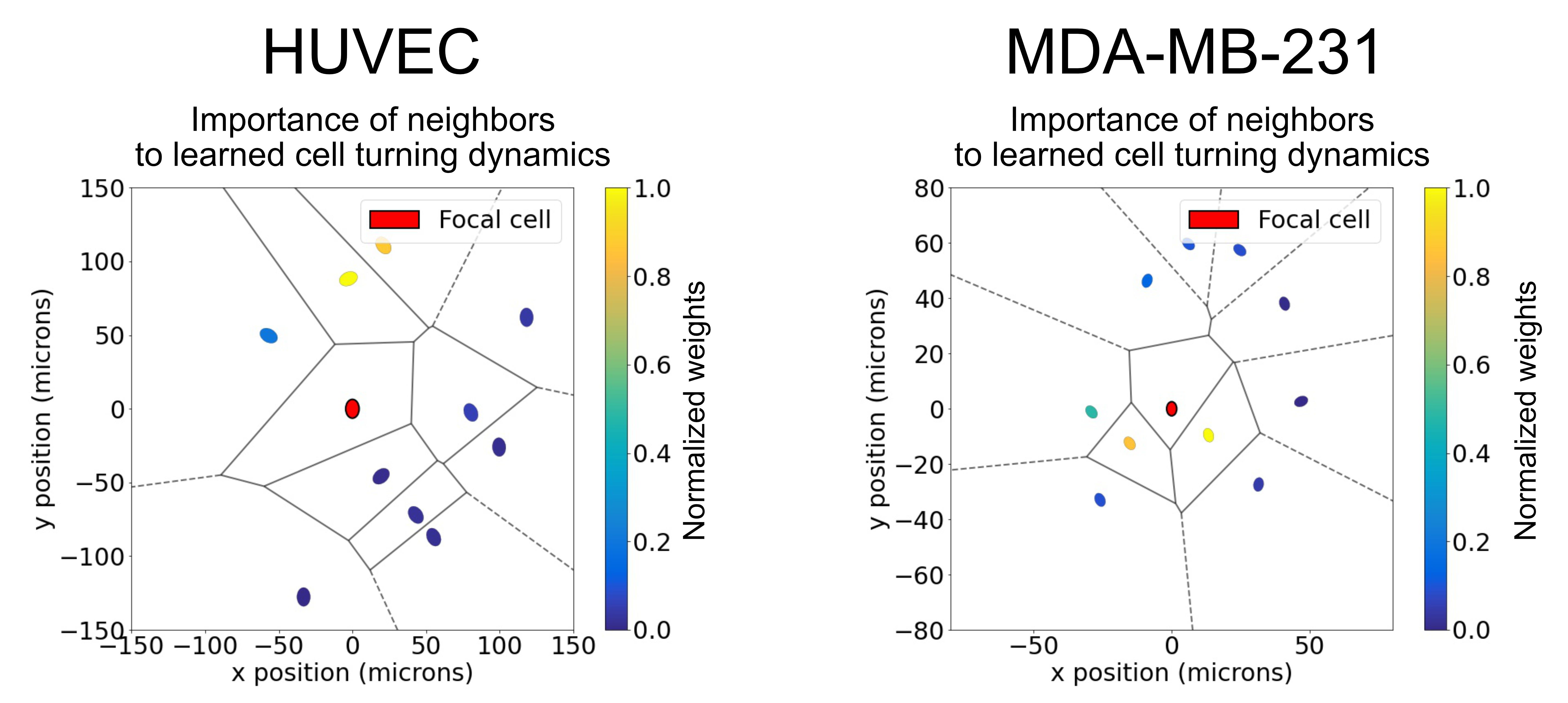

Supplement: S2 Fig — Individual agents are plotted in space (x, y) and colored according to relative attention weight (W) as in Eq 1 for HUVECs (left) and MDA-MB-231 cells (right). Cell position is representing using nuclei centroids and black lines indicate Voronoi cells (see Methods). (JPG) [file pcbi.1009293.s002.jpg]

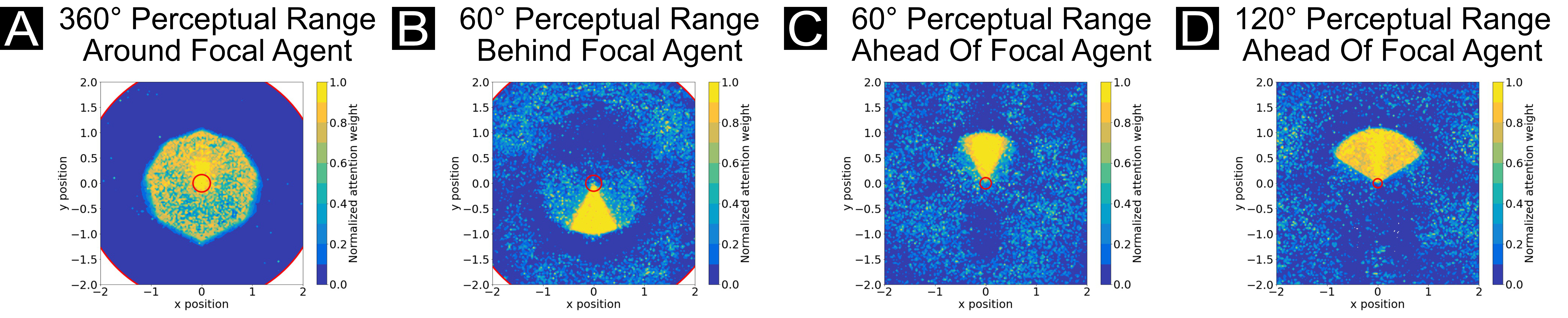

Supplement: S3 Fig — Attention maps for collective simulation (Vicsek model) Individual attention maps were produced for agent trajectories generated via (A) the classical Vicsek model with full radial perception, and Vicsek models in which the perceptual range between collective agents is constrained to (B) 60° (±30°) behind the focal agent, (C) 60° (±30°) ahead of the focal agent, and (D) 120° (±60°) ahead of the focal agent. The attention maps are able to capture these ranges directly from trajectory data alone. See Methods. (JPG) [file pcbi.1009293.s003.jpg]

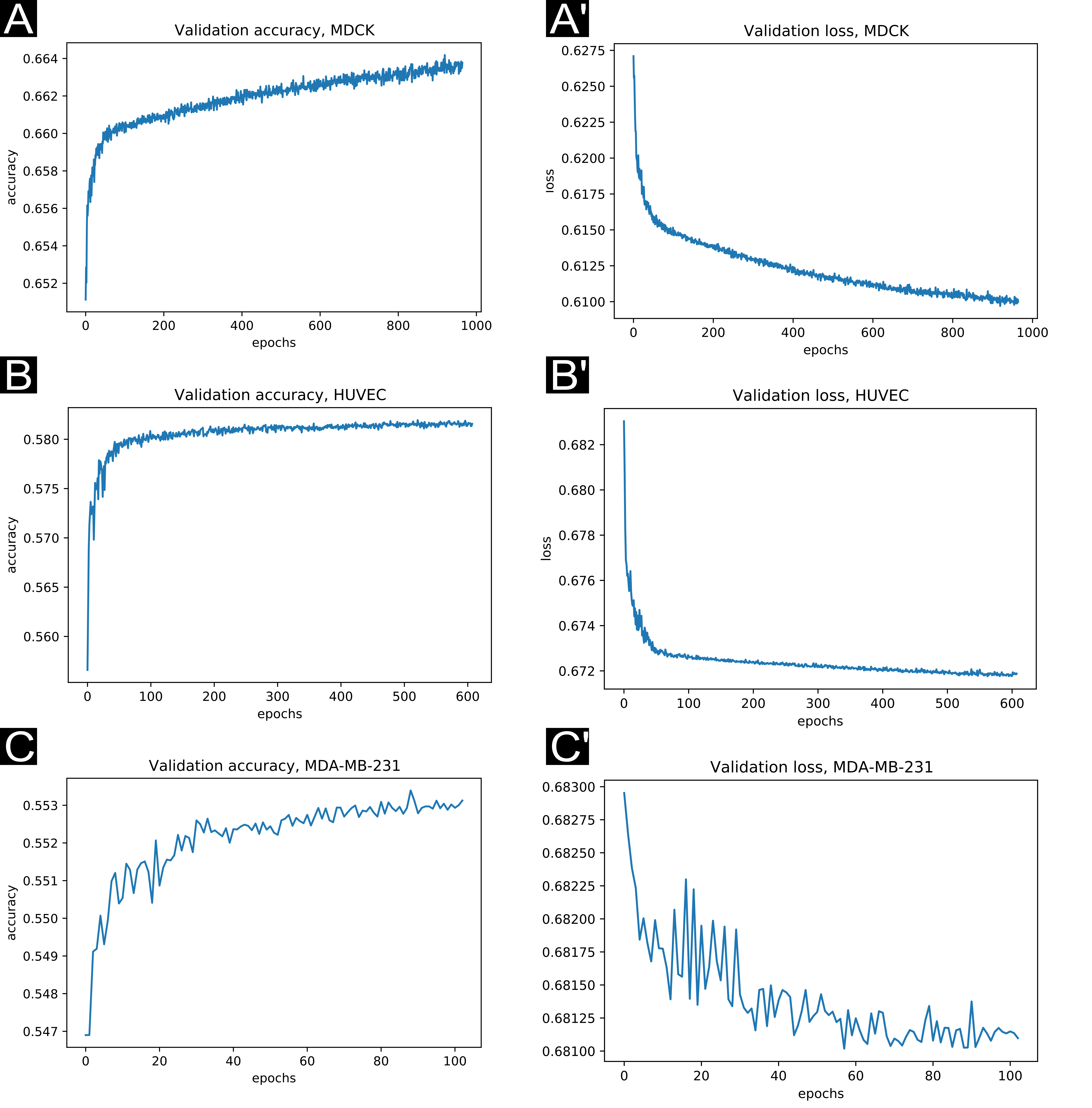

Supplement: S4 Fig — Early stopping was enabled, so that if the validation loss did not decrease within a set number of epochs, the training process was terminated. Validation loss was noisier when training the network on MDA-MB-231 data, in which there is reduced cell-cell coordination. (JPG) [file pcbi.1009293.s004.jpg]

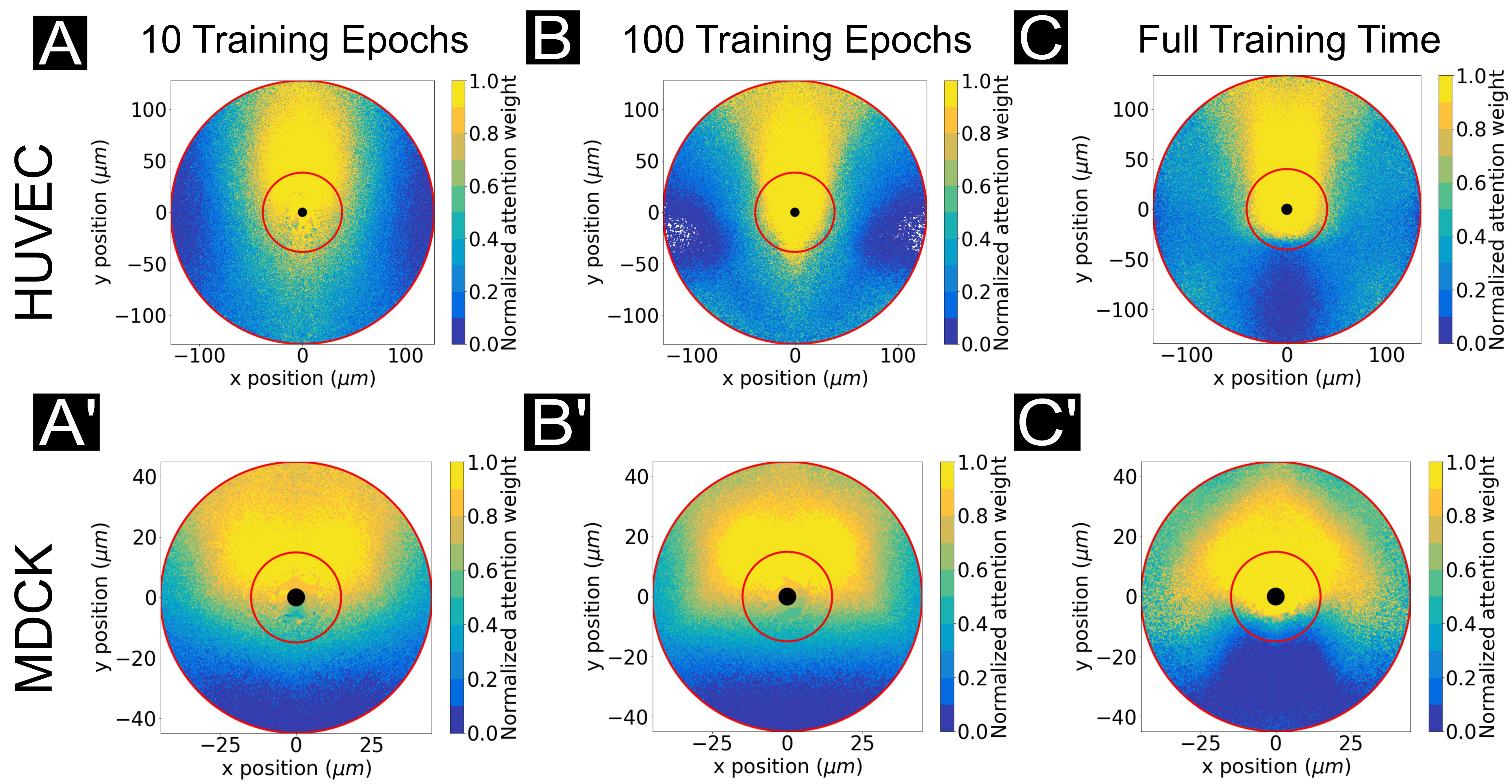

Supplement: S5 Fig — The attention maps for (A-C) the standard HUVEC cell system and (A’-C’) the standard MDCK cell system are shown, as (left to right) the number of training epochs is increased from 10 epochs, to 100 epochs, and finally to the fully trained system. The test accuracy for the HUVEC system after 10 epochs is 57.2% (57.3% large turns); while for the HUVEC system after 100 epochs it is 58.2% (58.7% large turns). The test accuracy for the MDCK system after 10 epochs is 54.1% (57.3% large turns); while for the MDCK system after 100 epochs it is 53.9% (56.9% large turns). (JPG) [file pcbi.1009293.s005.jpg]

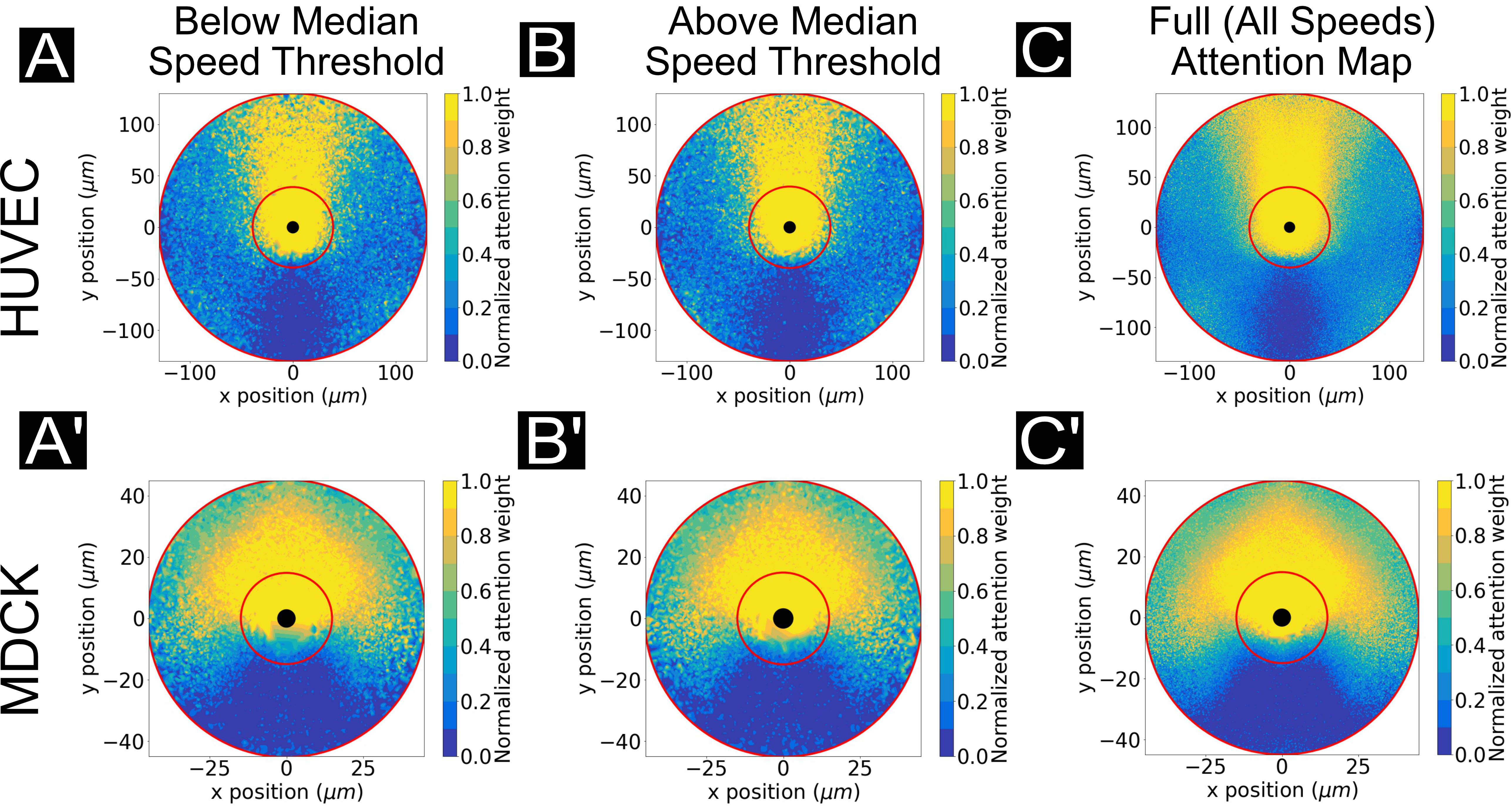

Supplement: S6 Fig — Attention maps are shown for the (A-C) HUVEC cell system and (A’-C’) MDCK cell system. We compare the full attention map for each system (C, C’) utilizing all available data points, to those data points where the focal agent speed is either (A, A’) below or (B, B’) above a threshold speed chosen to be the median speed value for all focal agents in the system. No meaningful structural difference was observed when speed thresholding was performed in this way. (JPG) [file pcbi.1009293.s006.jpg]

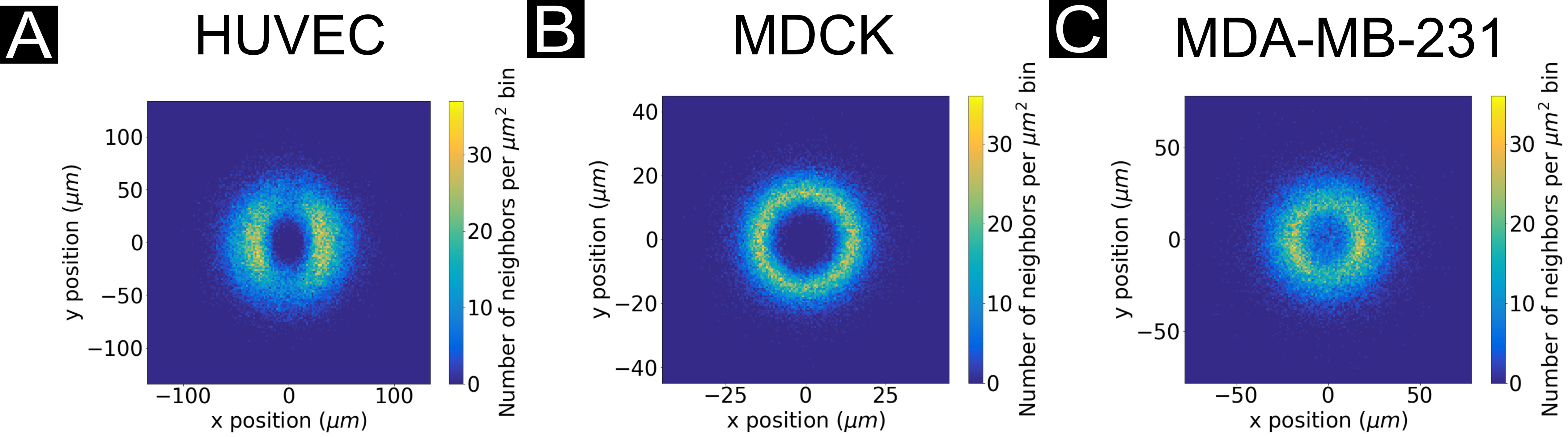

Supplement: S7 Fig — The histogram representation of the closest neighbor plots for (A) HUVEC, (B) MDCK, and (C) MDA-MB-231 cell systems are shown, analogous to the closest neighbor scatter plots represented in Figs 2E, 2E’, and 4C, respectively. (JPG) [file pcbi.1009293.s007.jpg]

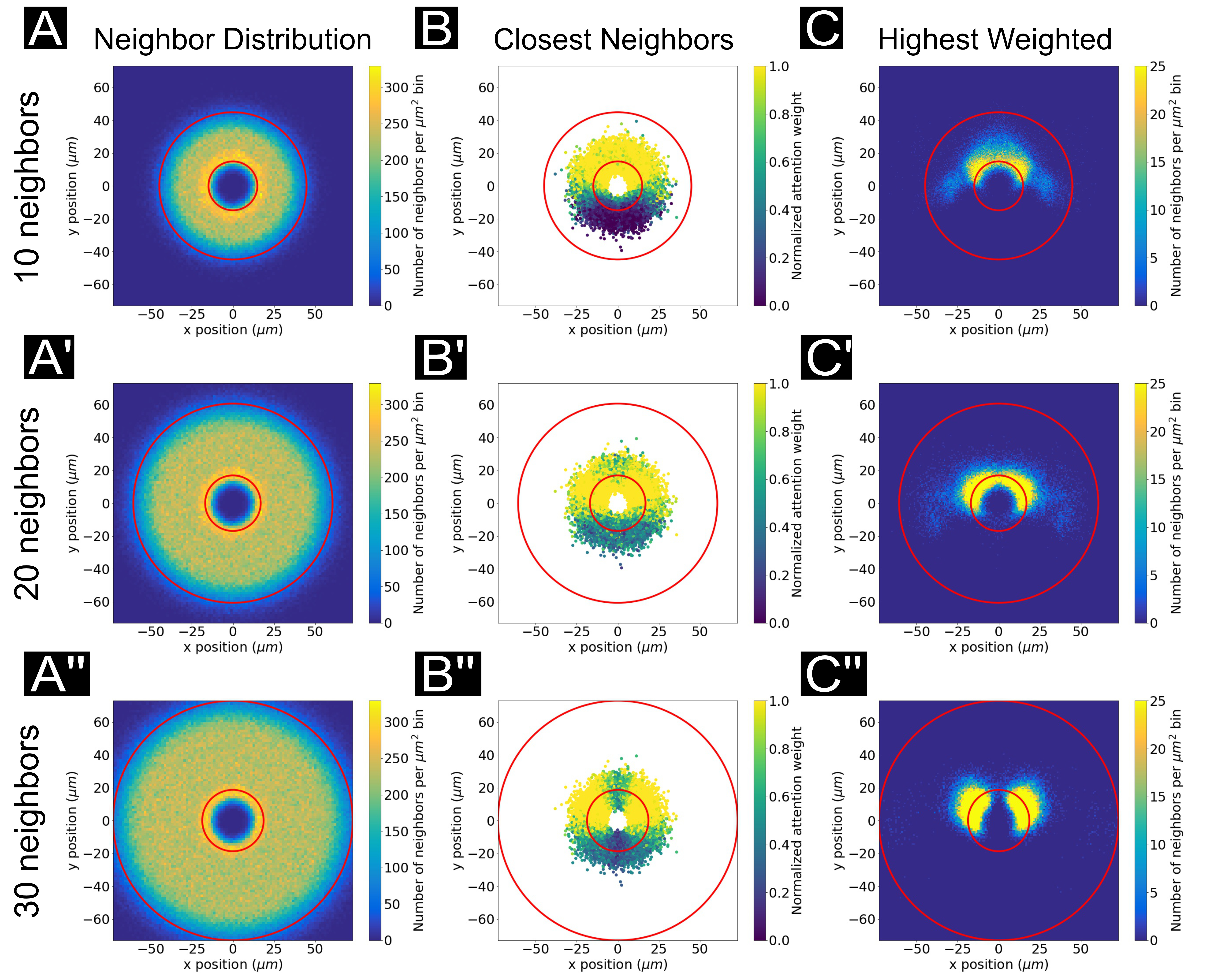

Supplement: S8 Fig — Plots shown are analogous to the neighbor distribution, closest neighbor, and highest weight neighbor maps shown in Fig 2D–2F’, yet corresponding to the 10, 20, and 30 neighbor networks with attention maps as in Fig 3D–3D”. (JPG) [file pcbi.1009293.s008.jpg]

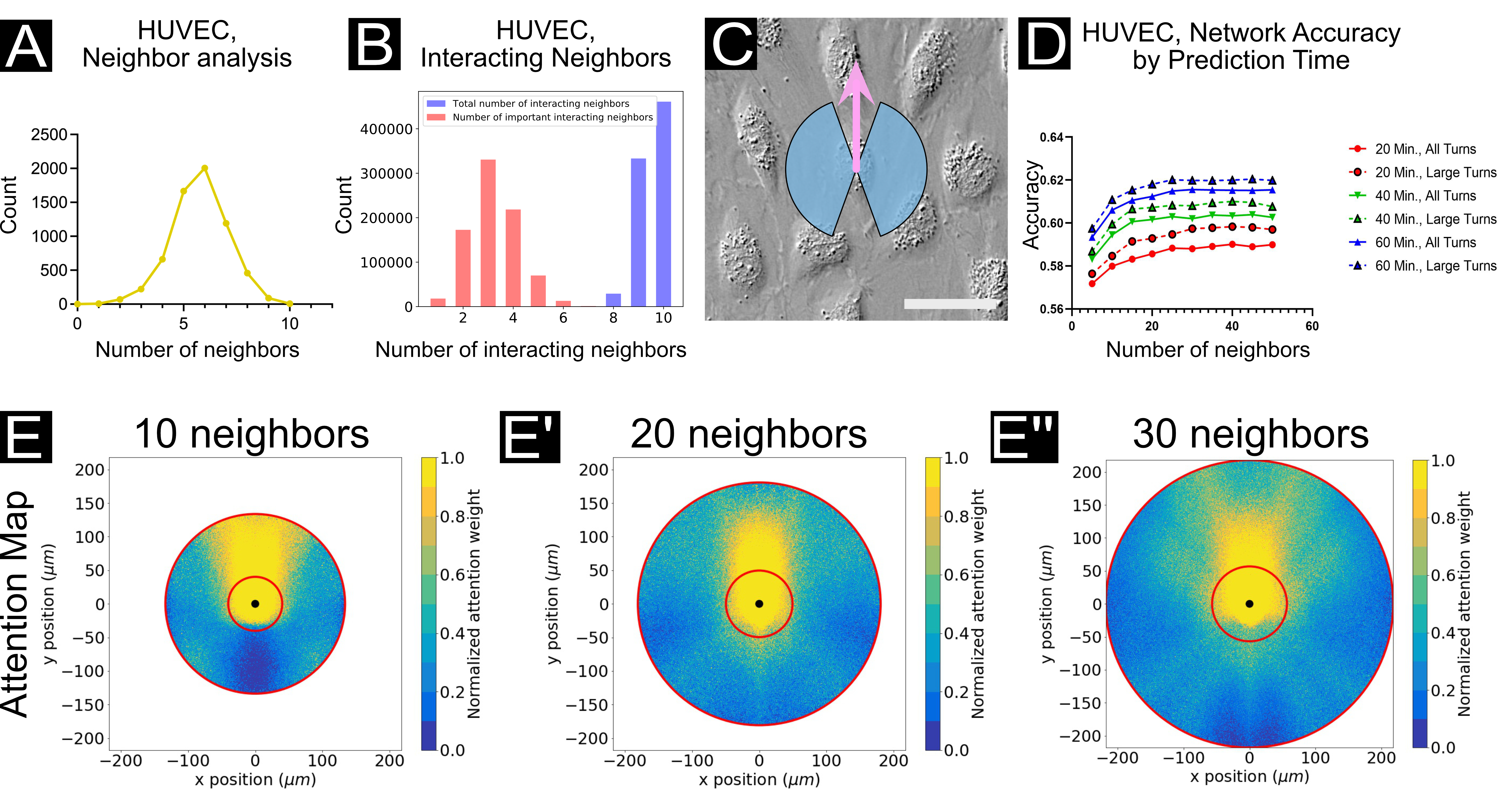

Supplement: S9 Fig — (A) The number of nearest neighbors based on an analysis of 1115 cells using the ImageJ/FIJI [47] BioVoxxel plugin[48] (see Methods). A peak can be observed at 3 nearest neighbors. (B) Histograms of total interacting cells (blue) and “important” interacting cells (red), as determined by a function utilizing the network aggregation weights (W) to estimate the most influential neighbors. (C) A snapshot of HUVEC cells with blue region indicating the extent of “large” turns (±20–160°) according to the focal cell trajectory (indicated by the pink arrow). Scale bar represents 20 μm (D) Network accuracy plots as prediction time and number of input neighbors is varied. Solid lines reflect accuracy scores for all turning angles in the focal agent trajectory; dashed lines reflect only large turns (±20–160°). Accuracy increases with both number of neighbors encompassed by the network and prediction time. Cell trajectory timesteps were fixed at 10 minutes. (E, E’, E”) Attention maps for networks encompassing 10 (left), 20 (middle), and 30 (right) neighbors. Plots shown here are analogous to plots shown in Fig 3, with cell trajectory timestep of 10 minutes. As the number of neighbors taken into consideration by the network increases, a wider spatial range of interactions may be considered for forward motion prediction. (JPG) [file pcbi.1009293.s009.jpg]

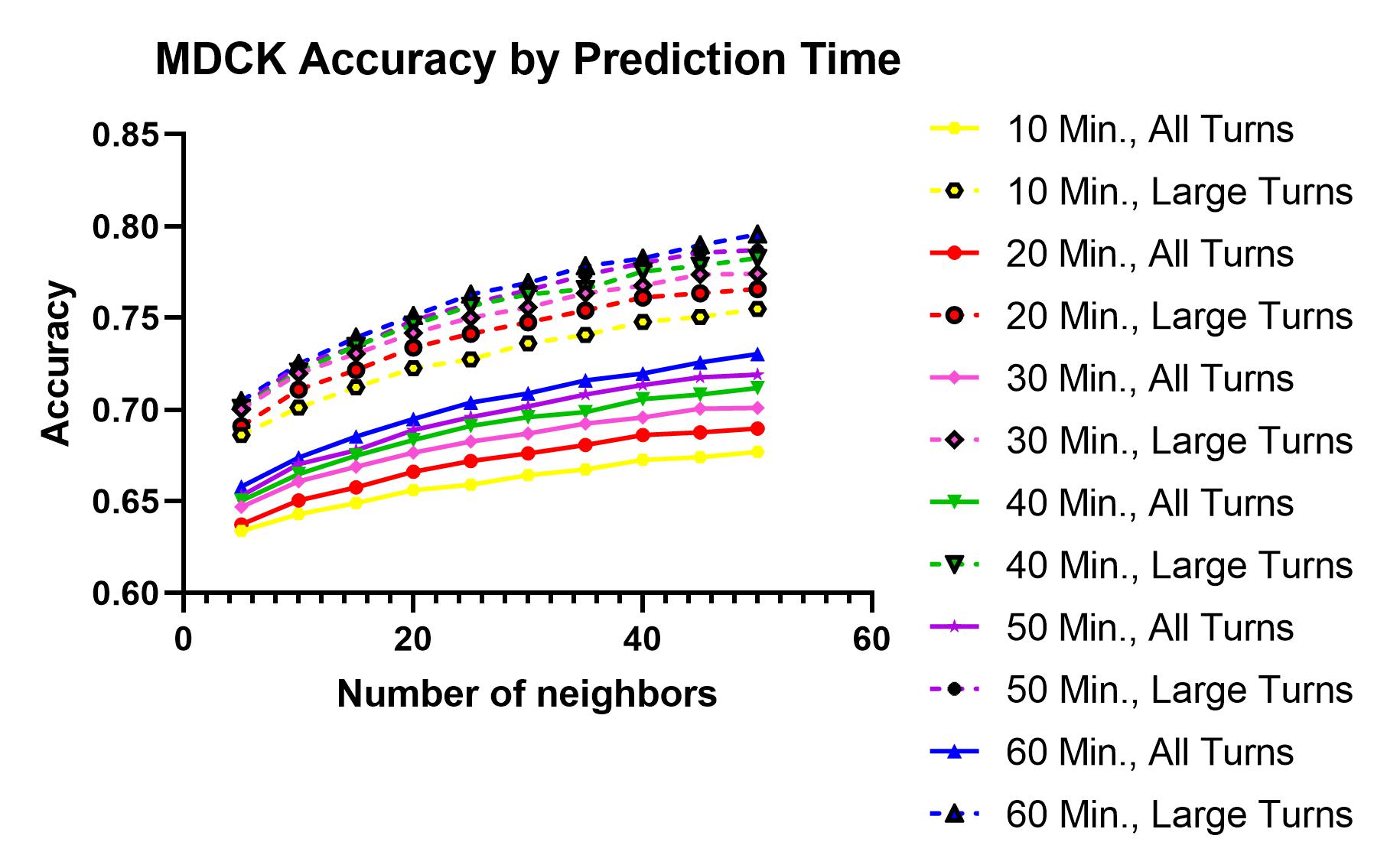

Supplement: S10 Fig — Network accuracy plots as prediction time and number of input neighbors is varied. Solid lines reflect accuracy scores for all turning angles in the focal agent trajectory; dashed lines reflect only large turns (±20–160°). Accuracy increases with both number of neighbors encompassed by the network and prediction time. Cell trajectory timesteps were fixed at 10 minutes. (JPG) [file pcbi.1009293.s010.jpg]

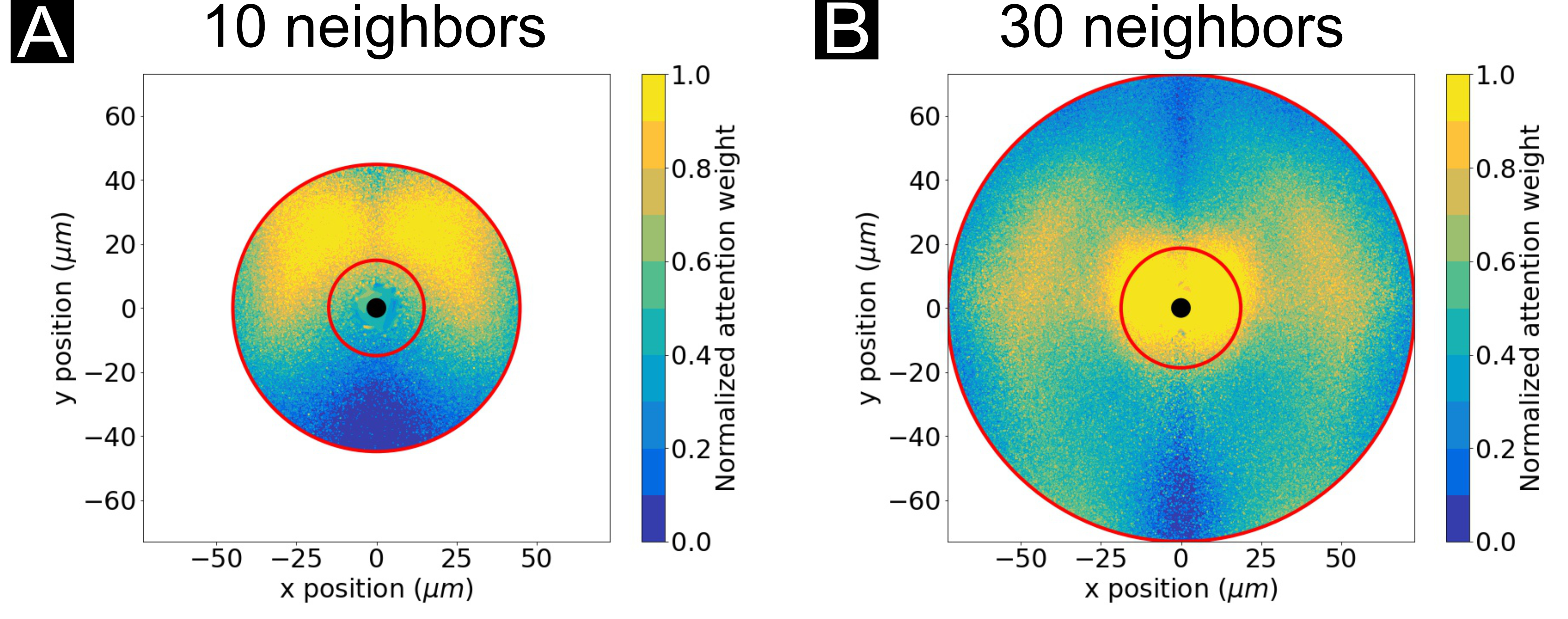

Supplement: S11 Fig — Representative attention weight contour plots are shown for MDCK cells with networks accounting for 10 neighbors in total (A) and 30 neighbors in total (30) with prediction time intervals of 60 minutes. For all conditions, normalized weight maps are shown and are analogous to the 20 minute prediction time interval attention maps shown in Fig 3D and 3D”. (JPG) [file pcbi.1009293.s011.jpg]

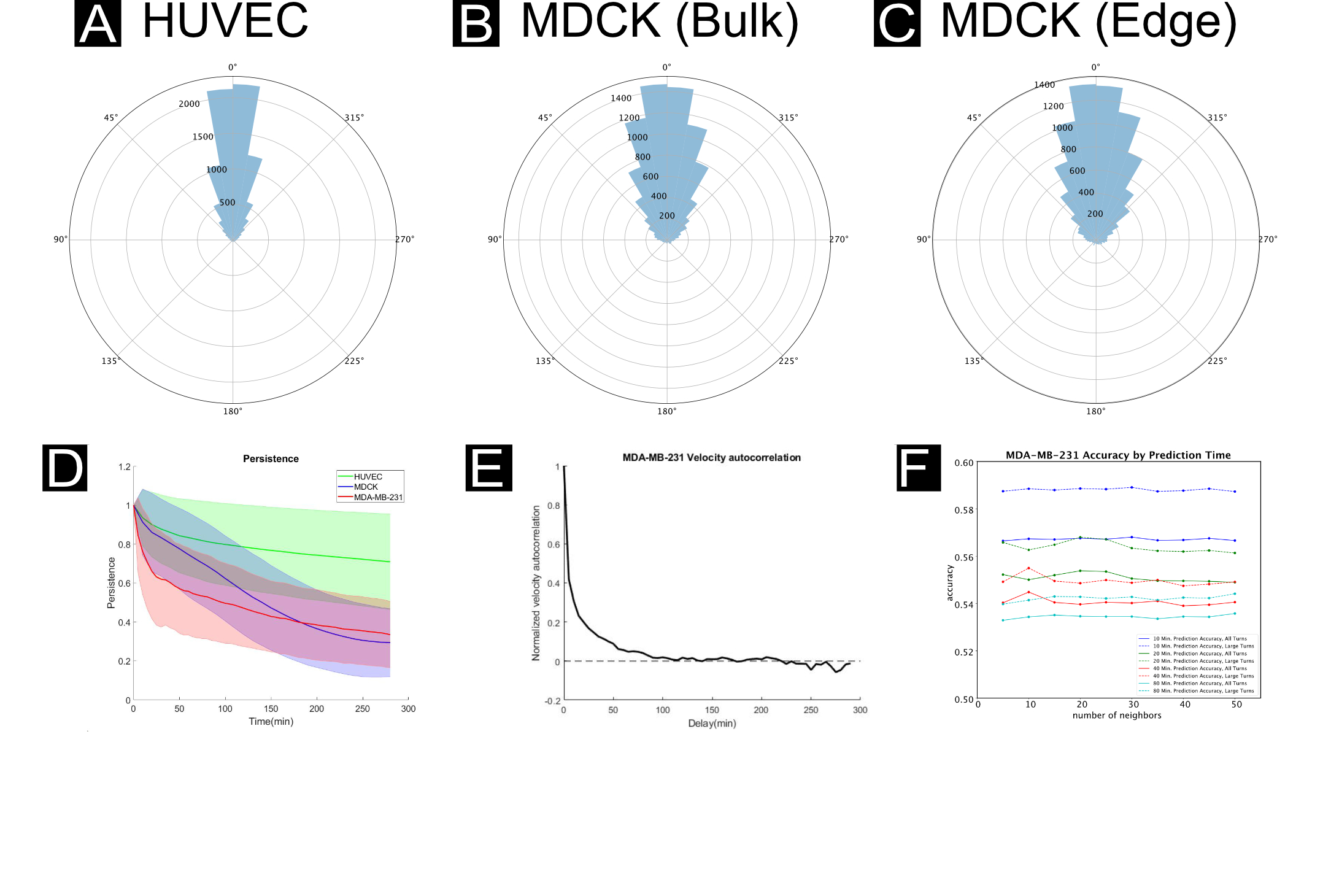

Supplement: S12 Fig — A radial histogram of turning angles from focal cell trajectories, shown for (A) HUVECs, (B) MDCK cells in the bulk region, and (C) MDCK cells in the edge region (from the same tissues; see Methods). HUVEC angles tend to fall closer to vertical (0°). (D) Persistence plot for all main cell systems indicating “directedness” by orientation over time. The persistence plot here highlights the tendency of the HUVECs in particular to proceed in a single direction; shaded zone represents standard deviation (see Methods). (E) Representative velocity autocorrelation for MDA-MB-231 cell system as an additional measure of the lack of dynamic persistence (generated using MSDAnalyzer). (F) MDA-MB-231 network accuracy is largely independent both of neighbor number and of time steps. (JPG) [file pcbi.1009293.s012.jpg]

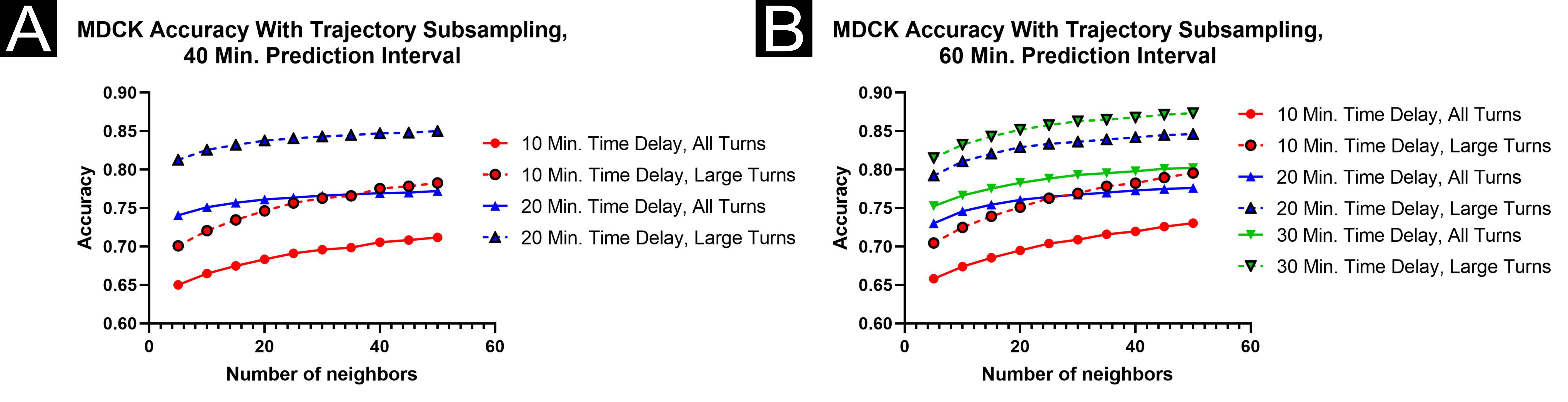

Supplement: S13 Fig — Network accuracy is shown as a function of number of neighbors encompassed by the network and time delay between cell trajectory points. (A) displays accuracy for a prediction time of 40 minutes, with 10 (blue) and 20 (green) minute time delays, resulting from subsampling of the initial trajectory results. (B) displays accuracy for a prediction time of 60 minutes, with 10 (blue), 20 (green), and 30 (red) minute time delays. Solid lines reflect accuracy scores for all turning angles in the focal agent trajectory; dashed lines reflect only large turns (±20–160°). Accuracy increases as time delay is increased; in this experiment, the same number of historical steps is utilized, so subsampled trajectories include data spanning longer total time intervals. (JPG) [file pcbi.1009293.s013.jpg]

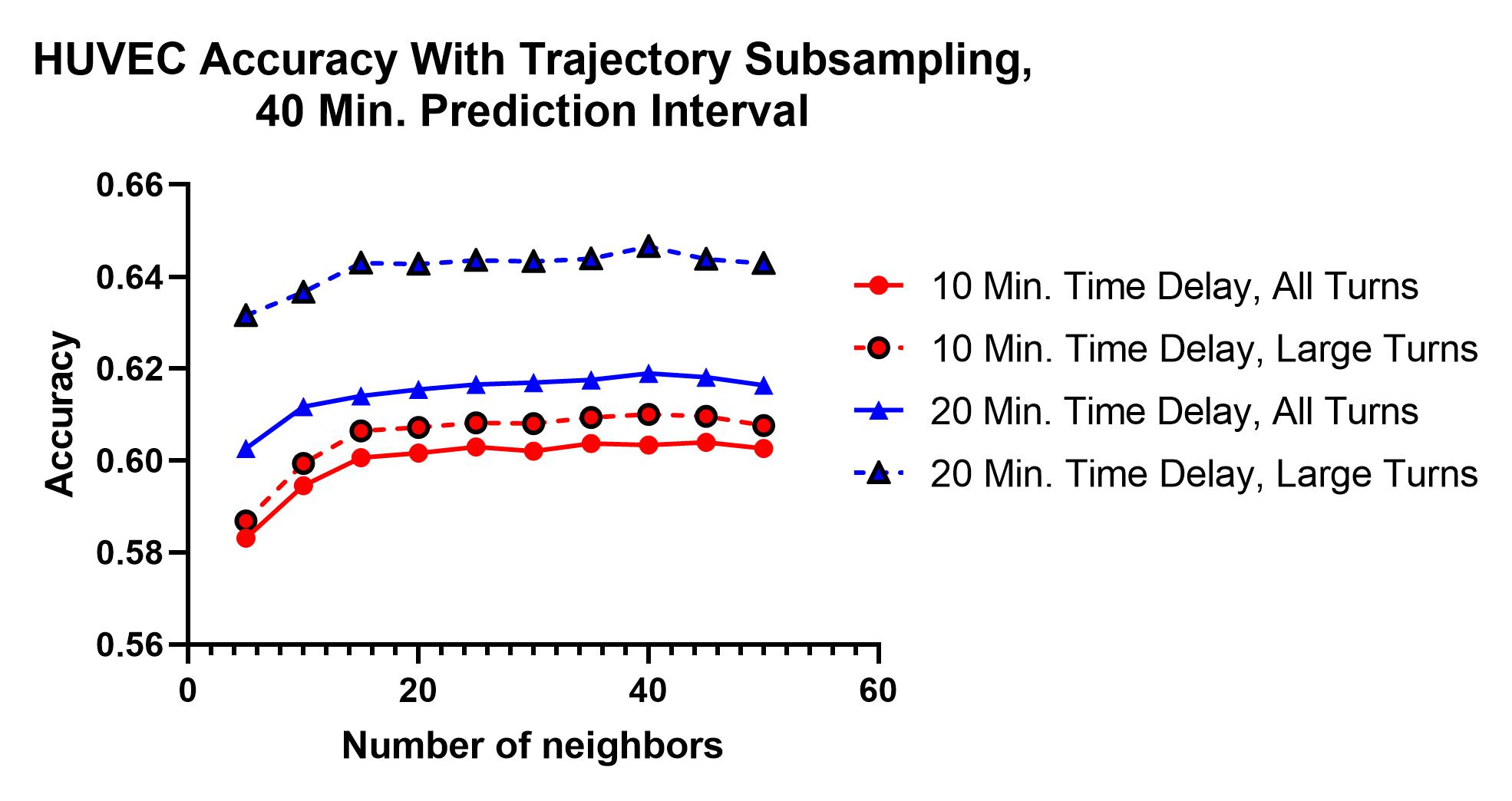

Supplement: S14 Fig — Network accuracy is shown as a function of number of neighbors encompassed by the network and time delay between cell trajectory points. (A) displays accuracy for a prediction time of 40 minutes, with 10 (blue) and 20 (green) minute time delays, resulting from subsampling of the initial trajectory results. Solid lines reflect accuracy scores for all turning angles in the focal agent trajectory; dashed lines reflect only large turns (±20–160°). Accuracy increases as time delay is increased; in this experiment, the same number of historical steps is utilized, so subsampled trajectories include data spanning longer total time intervals. (JPG) [file pcbi.1009293.s014.jpg]

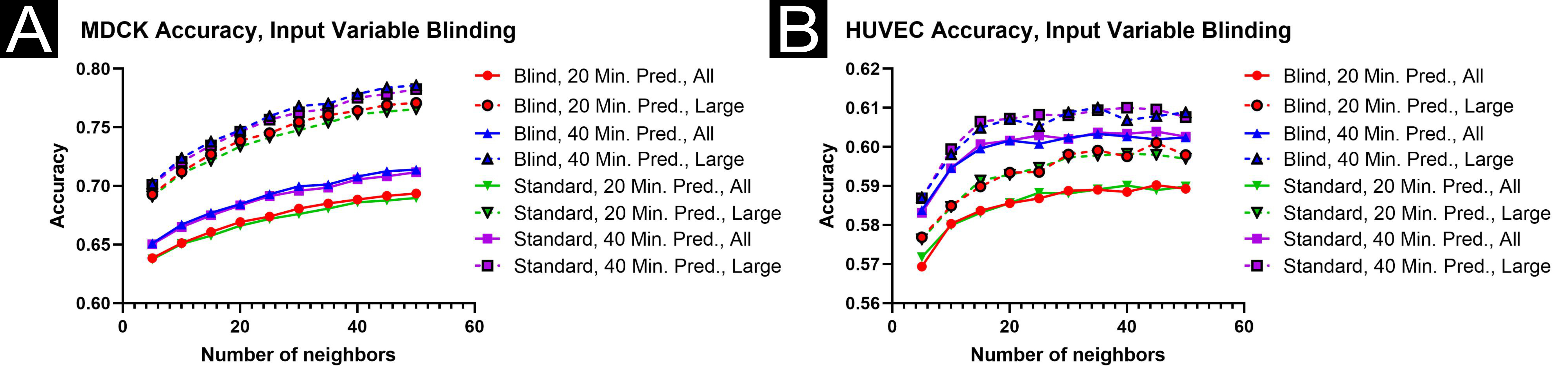

Supplement: S15 Fig — Network accuracy is shown as a function of number of neighbors encompassed by the network, prediction time, and input parameters to the network. Either the standard inputs are utilized (lighter colors, see Methods), or the model was blind to focal tangential acceleration and neighbor accelerations (darker colors; i.e., these parameters were excluded from model inputs). (A) displays accuracy for MDCK cells, (B) for HUVECs. Solid lines reflect accuracy scores for all turning angles in the focal agent trajectory; dashed lines reflect only large turns (±20–160°). Accuracy is not substantially changed as a function of acceleration blinding. (JPG) [file pcbi.1009293.s015.jpg]

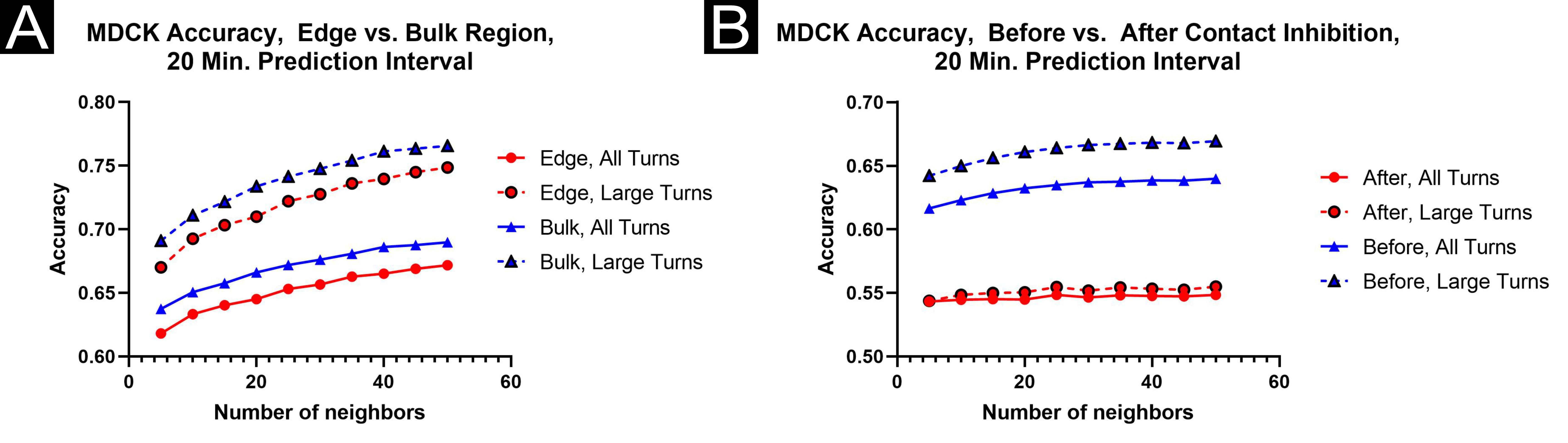

Supplement: S16 Fig — (A) Network accuracy plots as prediction time and number of input neighbors is varied for both bulk (darker colors) and edge (lighter colors) regions within a confluent MDCK tissue. Solid lines reflect accuracy scores for all turning angles in the focal agent trajectory; dashed lines reflect only large turns (±20–160°). Accuracy results tended to be slightly higher in the bulk region. (B) Network accuracy plots as prediction time and number of input neighbors is varied for the same MDCK tissues prior to (lighter colors) and after (darker colors) contact inhibition. Accuracy results were higher prior to contact inhibition. (JPG) [file pcbi.1009293.s016.jpg]

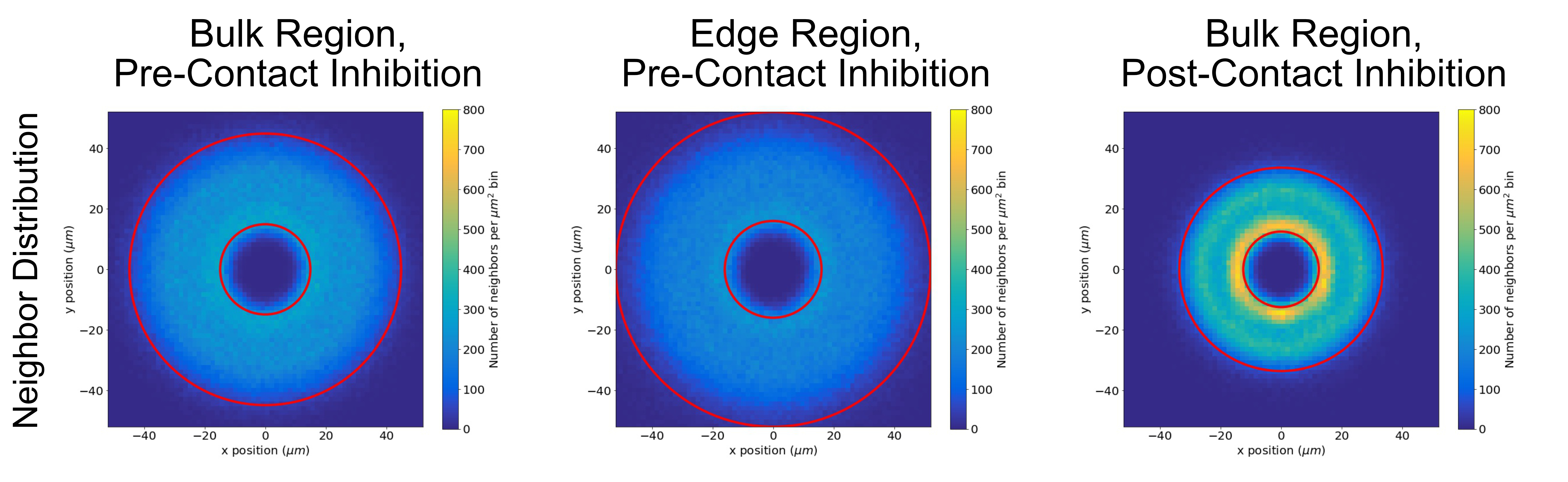

Supplement: S17 Fig — Histograms showing the distribution of data points (neighbor cell locations) from which the attention maps in Fig 5B,B’,B” were generated. (JPG) [file pcbi.1009293.s017.jpg]

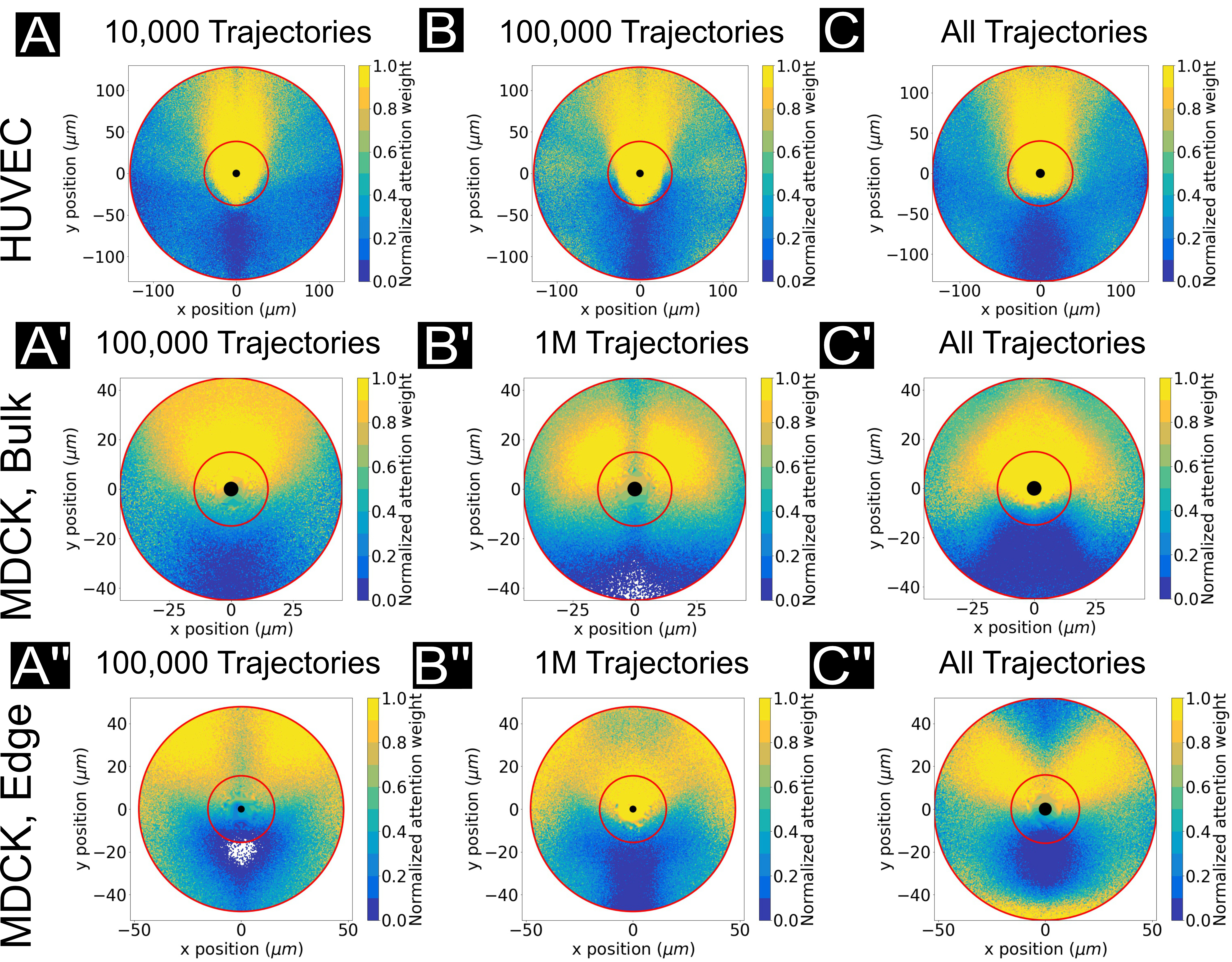

Supplement: S18 Fig — The training set size was reduced by limiting the number of total trajectories for (A-C) the HUVEC cell system (10,000 / 100,000 / 433,063 trajectories respectively); (A’-C’) the MDCK bulk region cell system (100,000 / 1,000,000 / 2,082,519 trajectories respectively); and (A”-C”) the MDCK edge region cell system (100,000 / 1,000,000 / 1,451,150 trajectories respectively). Accuracy results for reduced training set cases were as follows: For HUVECs, accuracies were (A) 59.0% (59.4% large turns) and (B) 59.2% (59.2% large turns). For MDCK (bulk region), accuracies were (A’) 66.7% (73.0% large turns) and (B’) 67.8% (74.9% large turns). For MDCK (edge region), accuracies were (A”) 66.1% (72.1% large turns) and (B”) 65.3% (72.1% large turns). (JPG) [file pcbi.1009293.s018.jpg]

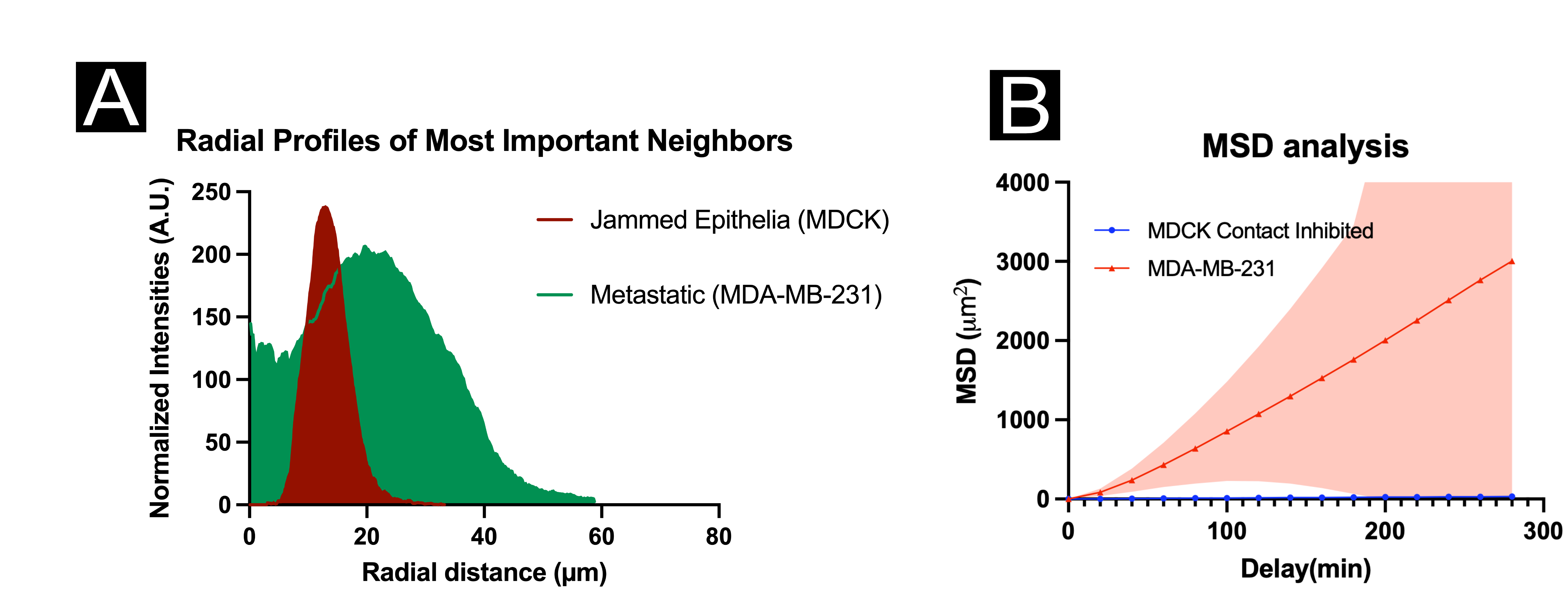

Supplement: S19 Fig — (A) Radial distributions of the most important neighbors is plotted for jammed MDCK tissue and MDA-MB-231 tissue. The most important neighbors of jammed MDCK are focused on ~10–20 μm zone while MDA-MB-231 tissue has a much broader distribution of the most important neighbors that also covers the focal cell, indicative of cells crawling over each other and a lack of repulsion. (B) MSD comparison between MDA-MB-231 and highly dense, jammed MDCK cells indicating how the MSD can complement the attention maps to reveal underlying differences. (JPG) [file pcbi.1009293.s019.jpg]

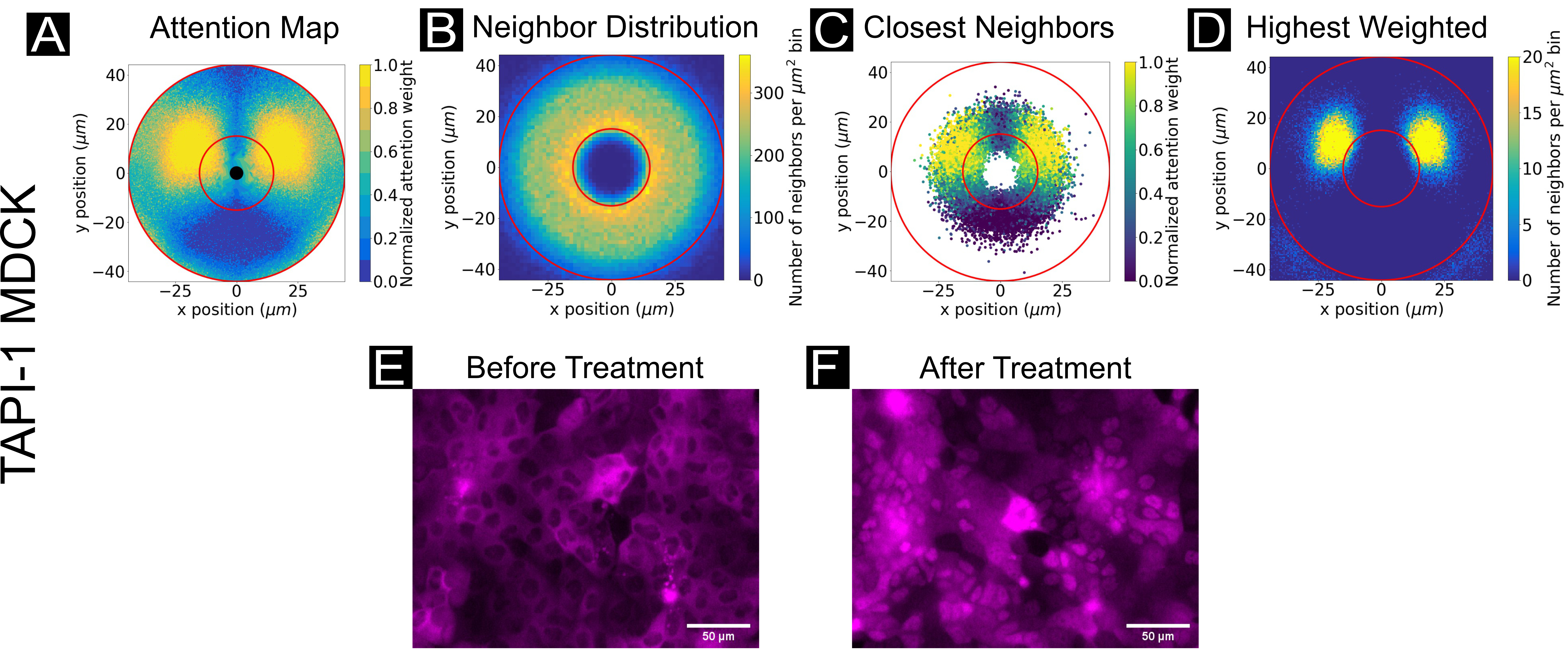

Supplement: S20 Fig — TAPI-1 was added to the standard MDCK cell system to inhibit cell-cell signaling (see Methods). (A-D) Plots shown are analogous to the attention map, neighbor distribution, closest neighbor, and highest weight neighbor maps shown in Fig 2C’–2F’. In comparison to the standard MDCK cell system, the attention maps reveal the loss of the relative influence of forward neighbors to the focal agent; however, “lobing” (relative influence of forward left/right agents) remains. The test accuracy was 68.5% for all turns, and 76.2% for large turns. (E-F) Representative images of MDCK cells immediately before and 2 hours after treatment with TAPI-1, respectively. Cells show lower ERK activity (higher nucleus intensity) after treating TAPI-1. (JPG) [file pcbi.1009293.s020.jpg]
