# Supplementary material for: Learning the rules of collective cell migration using deep attention networks
Source: PLoS Comput Biol. 2022 Apr 27;18(4):e1009293. doi: 10.1371/journal.pcbi.1009293 (PMC9106212; doi:10.1371/journal.pcbi.1009293)
Supplement: Attachment — Submitted filename: ResponseToReviewers.pdf [file pcbi.1009293.s025.pdf]

Reviewer responses to LaChance et al.
PLoS Comp Bio, 2021

Dear Colleagues,

Our responses to all reviewer comments are presented below in-line with the original comments. Our comments are indicated in blue, with text excerpts and line numbers provided where appropriate. We apologize for the delay, but the lead author graduated and took a new job part-way through the revision process, which necessitated bringing on a new team member to help finish the revision process.

Thank you for your consideration,

The Authors

Reviewer #1:

General Comments:

This is a particulaly interesting article at the intersection of machine learning and collective phenomena in biology. In my opinion, it certainly meets the bar for interest, depth, and creativity for publication in PLoS Computational Biology.

Congratulations to the authors on a very interesting and compelling piece of work. In my opinion, this article is of wide interest to a number of communities, and it's an example of precisely what PLoS Comp Bio can be publishing.

I have some questions and concerns that I would like the authors to address; these are all in the discussion section. I have an optional suggestion which would require more work; if the authors choose not to do this optional suggestion, I'd ask them to add a paragraph talking about the fact that this optional suggestion is a great idea and should be done by someone.

A note: the article is largely descriptive. Rather than test a particular hypothesis about the nature of cell navigation, their goal is to explore the different ways in which cells alter their trajectories in response to their social context. This is OK!

This is also a tool-confirmation paper. It is good that the cancer cells are messed up, because cancer is messed up. And I appreciate the ways in which the authors tie their descriptive findings to other knowledge in the literature. However, the finding that endothelial cells are NN while epithelial are long-range seems to be something we already knew (or should have been able to guess) -- it's the intuitively right answer, not an informative one. That's OK!

Major:

****** First, I have two requests for revision. These are just additions to the discussion.

1. to what extent are these attention networks capturing *causality*, rather than simple correlation? It is nice, for example, that these networks detect that the forward direction matters more than the rear (despite the existence of correlations to the rear). But what do we know about how these attention networks capture the causal effects? Are they just capturing the "more important" correlations, or is there something new in play? Are the attention networks using time, for example, as a proxy for causality (not a bad heuristic, of course!)

I don't expect the authors to have an answer here, but my suspicion is that this is just a more refined version of earlier correlative studies. Some discussion and analysis of exactly what's going on, even just in an exploratory mode, would be good in the discussion.

This is a good question! In this case, the results displayed in the attention maps are directly extracted from a learned model of the collective system dynamics. The deep attention networks are structured to predict the forward motion of the focal cell from its neighbors, as a function of various social and asocial input parameters. From this fixed model of the dynamics, the weight values corresponding to neighboring agents are plotted. Thus, the results are “causal” in the same way that the model is “causal”: and the extent to which those influence values correspond to the physical system will depend on the extent to which the model meaningfully represents the physical dynamics. So in this sense, we agree that time is essentially the proxy for causality, given the forward-predictive nature of the chosen model.

Text was added to the Discussion section to reflect this (lines 641+): “*Overall, attention maps can add new context and build on classical correlative or ensemble approaches, allowing for improved interpretability of collective motion dynamics. Fundamentally, the success of the intuitive power of the attention maps is a function of the success of the deep neural network model to capture agent-agent relationships within the collective, from which the learned, relative influence of each neighbor is obtained. Therefore, we can think of the learned relationships between agents as “causal” in that the learned model reflects real-world system dynamics.*”

2. it's not clear to me what the detection of long-range interactions means. As the authors note, the only direct physical coupling is to nearest neighbours. So what does it mean when the system is finding that long range matters more?

It seems there are (at least) two possibilities.

(A) there's chemosignalling; the chemical signals attenuate with distance, but there are more cells at longer range, and the second effect dominates. If this is true, then it seems like we're getting real biological information out of this process -- we're learning about the different signalling mechanisms.

(B) we're picking up epiphenomenal correlations; the nearest neighbour is setting the agenda, but it's doing so for both directions. The focal cell is correlated with the NN, the NN is correlated

with things forward, and so it looks like the focal cell is receiving instructions from longer distances.

Talking through these possibilities would give the reader a better sense for the biological insights that might emerge.

With the current network model structure, the model has complete information pertaining to the trajectories of all *n* neighboring agents. Therefore, we encourage those who wish to apply this method on larger groups of agents to be wary of non-physical results which may arise from having near-perfect information about the trajectory data of long-range neighbors. The objective of the neural network is to predict the future motion of the focal agent from social and asocial variables associated with itself and its neighbors. Therefore, while a physical cell may only take information from its immediate local neighbors (in the absence of chemosignaling and other long-range interactions), the network may be provided “hints” about future motion from longer-range bulk dynamics (e.g. a traveling front or long-range correlated motion). Yet, these effects are likely mitigated by carefully choosing network parameters, such as short trajectory times and, of course, smaller numbers of nearest neighbors to analyse the impact on attention maps. While chemosignaling may be picked up by the attention maps, it must be chemosignaling which occurs within the trajectory time span assessed by the network. The network should not be picking up on epiphenomenal correlations as described, since the network assesses the impact of neighbor cells on focal cells independently; thus, neighbors are weighted independently.

We have added some clarification in the section of the text pertaining to long-range interactions, as follows (lines 392-398): “*Again, we emphasize that the neural network will have access to trajectory data for each one of the n neighbors, whether or not the real focal agent does, and that long-range interactions (such as chemosignaling) can be captured as long as they occur within the timespan of the trajectory data. Users must be wary of any unique boundary phenomena (sustained tissue outgrowth and moving fronts), which may be captured within the analyzed timeframe and can influence the learned importance of long-range neighbors.*”

****** And, I have one suggestion for the authors to investigate: what happens with simulated data? Let's say that you create a fake tissue, in silico, with cells that are following nearest neighbour rules (or have some other attention kernel).

Can you recover that kernel? How well can you do so? What do you get right about the kernel? For example, can you get accidental long-range attention windows even when the underlying dynamics are nearest neighbour? I don't think this needs to be exceptionally long.

A simple in silico experiment with two different navigation rules (one NN, one more long-range, perhaps roughly matching the HUVEC and MDCK cases), and the results of the attention network method, would be enough.

I think this could really help make the paper more widely compelling for readers, in part because most people are not going to go through the trouble of reproducing the analysis.

I don't want to say this is a mandatory edit, because I think it is a lot of work. However, if the authors choose *not* to do this, then they need to add a paragraph talking about how they *didn't* do this analysis, why it's a good idea, etc. -- just punting to future work, but making it clear that it's a question in play.

We want there to be some match between inference and reality, and a simulation is a nice way to check that things aren't going off the rails. Without that check, questions remain about the ways in which things could go wrong (e.g., remark #2 above.)

This is a good point and we agree that a robust validation process is essential to trust the model. Fortunately, part of our original confidence in the model comes from it having already been validated against a boids-like simulation in Heras et al. (see Fig. 4 in that paper). However, as both Reviewers #1 and #2 suggested validating against a model, we have added an additional suite of model validation data based on the Vicsek model, as suggested by Reviewer 2. In this model, agents move with constant speed and adjust their heading to the average of all other agents within their perception area, defined by a circle of chosen radius. We tested the network on simulations produced using the Vicsek model as well as simulations from a modified Vicsek model, where each agent’s perceptual zone is restricted to a chosen angle. The network recovered these chosen perceptual angles completely. This was quite encouraging as these underlying rule changes are impossible to detect by eye and very difficult to extract using classical methods.

The main text has been updated to reflect these changes (lines 111+):

“To first build confidence in this approach from complex collective migration systems, we tested network performance against the classic Vicsek agent-based model of collective motility. Here, agents move with constant speed and adjust their heading to the average of all other agents within their perception zone, typically a circle of a given radius, and we implemented this in a manner that allowed us to directly pass trajectory data of individual agents to the attention network (see Methods for our simulation parameters and approach). First, we confirmed that the network could recover the largely radially symmetry attention zone of the classic Vicsek model (S3A Fig). Next, and more striking, we implemented specific narrowed perceptual zones, reducing any given focal agent’s awareness to a small sector of different widths and directions. To a human observer, this subtle shifts in perceptual zone are impossible to detect by observation alone, and would be quite difficult to extract using classical methods. However, the network was able to accurately recover each unique perceptual zone we tested (S3B-D Fig). Together with boids model simulation results in Heras et al. [9], these data validate the efficacy of attention networks and allowed us to move forward with cellular analyses.”

The *Methods* section has been updated to describe the simulation test (lines 810+):

"Collective Simulation Analysis

To validate if deep attention networks recover differences in attention in known cases, we trained them using simulated trajectories. This data was generated using a commonly used model for collective motion - the Vicsek model. The model was set up according to the original paper[58]. The parameters used are as follows: $\eta = 0.1$, $L = 50$, $N = 3000$, $r = 1$, $v = 0.3$, $t_{MAX} = 200$, $\delta t = 1$. For some simulation cases, changes were made to the model in order to reduce the perceptual zone of each agent. In the modified Vicsek model, a focal agent's heading will only be affected by other agents within its perceptual zone. We tested four cases defined by the agents' perceptual zones: full 360˚ perception, 60˚ perception in front of the agent, 120˚ perception in front of the agent, and 60˚ perception behind the agent. Each dataset contained 15 simulations in the training set and 3 in the test set. The networks were trained using 15 nearest neighbors and 1 prediction time step."

Finally, S3 Figure has been added to the text as a Supplemental Figure.

Minor:

None

Reviewer #2:

General:

In this manuscript, LaChance et al. present an approach to learn aspects of the interactions between cells based on deep attention networks directly from experimentally measured cell trajectories. Specifically, trajectories of monolayers of different cell types (HUVEC, MDCK, MDA-MB-231) are passed through a deep attention network to identify the relative importance of neighbouring cells (quantified by their weight in the network) to predict the turning behavior of any given cell in the monolayer. Here, the authors largely follow the methodology of ref 9 in the paper. With this approach, the authors infer "attention maps" which give the average importance of neighbours as a function of polar angle from the considered cell. Interestingly, the different cell types have different attention maps: HUVEC cells have much more focused maps, indicating larger importance of head-neighbours. In contrast, MDA-MB-231, which are known to have a more random migration phenotype, have completely isotropic attention maps. While this approach is new to cell migration research and interesting, these findings are not interpreted or put into context with existing cell migration literature.

Overall, this study presents a new way to analyse cell migration based on trajectory data, which could present an important tool in the future. However, the method is not tested convincingly on benchmark data, where the interactions are known, and it is unclear what new insights are gained from applying it. Most importantly, the attention maps are inferred, but then never interpreted in depth. Thus several questions remain unanswered: What new things have we

learned about cell migration with this analysis? Why should others apply this method in the future? What is the advantage of this method over existing analysis and inference methods?

We appreciate the summary and have addressed the specific points as they are raised below.

Despite these shortcomings, I believe that with revisions, the paper could be suitable for publication. Specifically:

Major:

- The paper lacks a discussion of the biophysical implications of the findings. An interesting aspect of the attention maps is that many collective cell migration models based on active particles assume a radial symmetry of the interactions: cells interact with forward neighbours just as much as with backward or lateral neighbours (see e.g. 10.1371/journal.pcbi.1002944 ; 10.1073/pnas.1219937110 and many other papers that implement alignment interactions inspired by the Viscek model). Such interactions can also be inferred directly from cell trajectory data, without relying on machine learning (see https://doi.org/10.1073/pnas.2016602118). The findings in this study seemingly contradict this assumption - however it is not clear whether they really contradict it, or whether this is an artefact of the method. Would an attention map for a Viscek-like model for cell migration in the flocking regime that correctly identify the radial symmetry of the interactions? In this parameter regime, the model should capture the cell data at the level of MSD, velocity cross correlations, and order parameter, but it would still be based on radially symmetric interactions. But, the attention maps should still correctly infer the radial symmetry. Then, using a model where these interaction in fact depend on the angle from the cell, the attention maps should also infer this angle correctly. **Only if this is the case can the attention maps really be used to learn something new about cell migration. If it is not the case, then it is unclear how to interpret attention maps.**

Model validation is an important step, especially in the context of existing physical and biophysical active matter approaches as noted by Reviewer 2. As this is quite important, we addressed it explicitly as the reviewer suggested using the Vicsek model itself as this should be salient to the pan-physics community. We do note that we also reached out to the authors of several of the suggested papers but they were unable to provide their computational models of cell migration for us to test. Overall, we specifically validated both the radial symmetry of the standard Vicsek model and that our network could accurately detect unique perceptual zones implemented in a modified Vicsek model, and these results are summarized in S3 Fig and discussed in the text. Please see our response to a similar question from Reviewer #1 (final major revision request, above) for further detail.

- In the introduction there is a lot of discussion about velocity cross correlations between cells, yet this quantity is never presented for the data. Could the authors show the velocity cross correlations for the 3 cell types in Fig. 1? Both the mean speed and the MSD are really single cell statistics and don't quantify collective motion.

We agree. We added velocity cross correlation graph on Fig.1 E and describe higher velocity cross-correlation on HUVEC and MDCK cells compared to MDA-MB-231 cell. (Line 30+)

- the language around attention is used very loosely in the paper (e.g. line 59, 104), and often seems to suggest that the cells really "pay attention to their neighbours". There is no notion of this in the literature and so it would have to be defined carefully. The wording should be much more careful to not mix up animal systems like fish with unconscious systems like cells.

We agree, and have worded key portions of the text more carefully to discuss attention in a cellular sense. While we still use the technical, established terminology of ‘attention maps’, we have taken care to better describe these in the cellular context as relating to ‘relative influence’ rather than conscious attention processes. For example, we have modified the text in the following places:

(Lines 63+): “*Deep attention networks trained on cell trajectory data can directly reveal new types of collective information, such as the learned relative influence of neighboring cells* ***to forward and turning motion of a focal cell****.*”

(Lines 83+): “*Specifically we ask the following question of the deep attention network: given a ‘focal’ cell in a group of cells of a given type, where the ‘focal’ cell is simply the primary cell of interest and interacts with n nearest neighbor cells, to which other cells does the focal cell seem to “pay the most attention” when deciding how to turn? More technically: which neighboring cells have greater relative influence on the forward motion and turning dynamics of the focal cell, according to the dynamics learned by the model (Fig. 1F)?*”

(Lines 108+): “*thereby allowing us to determine for any given cell which neighbors most strongly influence the future motion of the focal cell according to the trained deep attention network model (Fig 1F, S1).*”

(Lines 142+): “*but relating the ensemble visual patterns to which neighbors are most influential to the future motion of a given focal cell, as a function of the learned dynamics, is not simple.*”

(Lines 147+): “*the question we posed above about how the dynamics of a given focal cell are influenced by specific nearest neighbors.*”

- similarly, the paper would benefit from a discussion of how the concept of attention can be interpreted in the context of cell migration: how does it connect to the concepts usually invoked in the literature like active particle interactions, traction forces, monolayer stresses, ...

This is important. We’ve now added additional context now, primarily at lines 628+ as described below: *Broadly, attention analysis reflects the integrated effects of a variety of cell-cell coupling mechanics such as traction forces, cell-cell junctions, jamming, and chemical signaling[52–54]. While attention maps cannot deconvolve these effects, they can still highlight the resulting*

phenotypes. Extending the earlier discussion, the powerful forward neighbor influence in HUVEC attention maps derive mechanistically from the polarized VE-cadherin structures (Fig. 2) that generate front/rear tension with no lateral coupling[40]. Similarly, the shift in attention maps with young versus old MDCK epithelia reflects the classic biophysical jamming transition, while the distinct influence pattern in attention maps taken at the growing edge of epithelia likely reflect the unique traction force and monolayer stress states at epithelial boundaries. Attention mapping may eventually help to connect biophysical mechanisms to collective behavior 'rules'.

- lines 102-104: the authors argue against ensemble analyses, but then proceed to generat ensemble averaged attention maps. What do they mean with ensemble analyses? The arguments that follows is not convincing: why are ensemble averages not informative about interactions?

The text was updated to describe "Classical ensemble analyses" instead; here, we emphasize that we **complement** classical ensemble methods which cannot on their own distinguish the contribution of individual neighbors to the extent that the attention network can, nor can they produce such interpretable visualizations.

- Fig 4E: isn't this a trivial result for trying to predict a persistent random walk with a deterministic model? How does the prediction time interval compare to the persistence time of the cells (calculated e.g. from MSD or velocity autocorrelation)?

Yes, the drop in accuracy should be trivial for a system with dynamics as a persistent random walk, and this is why we displayed this plot originally. To highlight the relationship between the persistence, we have included additional subplots in supplemental figure S12 Fig., clarifying in particular the randomness in the MDA-MB-231 system with an additional velocity autocorrelation plot. We also clarify this in the text (lines 506+):

"*The velocity autocorrelation (S12E Fig.) plot drops off sharply within approximately 50 minutes, which is consistent with the drop-off in accuracy within the first approximately 50 minutes in accuracy vs. prediction time interval, as the system loses its dynamic 'memory' within this time interval.*"

- the abstract is very confusing: there is a wealth of literature on expressing cell migration rules in interpretable form (e.g. everything on Contact Inhibition of Locomotion, alignment interactions etc.). The concept of a focal cell is not explained. Attention is not defined, and must be as it's a new concept for cell migration research.

We have modified the abstract for additional clarity, in the following phrases:

"*...yet it has been difficult to simultaneously express the 'rules' behind these motions in clear, interpretable forms that effectively capture high-dimensional cell-cell interaction dynamics in a manner that is intuitive to the researcher.*" (line 2-4)

"*to the learned turning decisions of a 'focal cell' – the primary cell of interest in a collective setting. Colloquially, we refer to this learned relative influence as 'attention', as it serves as a proxy for the physical parameters modifying the focal cell's future motion as a function of each neighbor cell.*"(line 10-13)

Minor:

- In line 4-5 the historical treatment seems to contradict the dates on the cited papers: were velocity correlations really first used on animal data and then "repurposed" for cells?

Good point: this line has been updated to better reflect the history of the field, with an additional reference and text modification (lines 26-27), *"in bird flock and fish school dynamics[10–12]"*.

- In Fig 1E, the massive green area is not labeled or explained. The MSD should in addition be plotted on a loglog scale, to back up the claims in lines 9-11.

We agree; we included both natural scale and log-log scale plot on S1 Fig. We shifted the MSD to the supplement in favor of the velocity cross-correlation in Fig. 1, and also explained the shaded green area in the caption to the supplemental figure. Briefly, the shaded zones reflect the weighted standard deviations of the MSD trajectories (see Methods and the manual for the MSDAnalyzer package).

- line 132 should say experimental model systems rather than models to avoid confusion

We agree; the text was updated in line 177 exactly as recommended.

- line 205 figure reference seems to be mixed up

We agree; text in line 261 now reads "*as a function of radius (Figs. 2C-F').*".

- line 313 what is the logit boundary? is it defined anywhere?

We clarify this meaning in the text in two places. First, via an addition to the introduction: (lines 100-102): "*The logit differentiates between forward motion in the left hemisphere with respect to the focal agent's forward heading, and forward motion in the right hemisphere.*"

Then, in the line referenced here:

(lines 370+): “*at the logit boundary (directly forward of the focal agent) where small spatial variations may produce a change in left vs. right turn classification*”

- the concept of a focal cell could be better introduced (it's just the cell that's being focused on, it's not a special cell within the monolayer like a leader cell)

Text was added to better explain this term (lines 84+): “*given a ‘focal’ cell in a group of cells of a given type, where the ‘focal’ cell is simply the primary cell of interest and interacts with n nearest neighbor cells,*”.

Reviewer #3:

This work focuses on applying a deep attention network developed by Heras, et al. to coordinated cellular migration to compare behaviors across cell types. The parameters controlling coordinated cellular migration are challenging to intuit from existing data, and deep learning offers an unbiased approach with no assumptions to developing new metrics to interpret data about multicellular migration. The manuscript is very clearly written and transparent about the advances and current limitations. However, we have several concerns to be addressed before publication.

Major:

Innovation, originality, and importance to the field are publication criteria for PLoS Computational Biology. This manuscript could be strengthened by a novel finding instead of applying an existing method that largely confirms what is known. This could go to the extent of examining multicellular migration in an understudied cell system. However, addressing the other major concerns could also address this concern, and so we do not want to prescribe a specific way of addressing this concern.

Although originality is not essential to this style of publication, we do agree that the analysis of an additional modified cell system strengthens the paper - refer to next comment.

How extracellular signaling impacts the model assumptions is unclear. The work appears to assume that physically-linked cells are the only important ones for attention maps, but cells such as MDCK cells are known to release EGF during collective migration. Perturbing extracellular signaling with flow and testing the model's response could be an exciting test of the limits of this approach.

We agree that analyzing a system in which cell signalling via EGF is perturbed is interesting and important. We chose to modify one of our canonical cell model systems, MDCK cells, with a drug, TAPI-1, which inhibits spatial signaling and extracellular signal-regulated kinase (Erk)

activation. We specifically chose TAPI-1 in consultation with the Toettcher Lab at Princeton who specialize in EGF and Erk manipulation. They had advised against using flow specifically as it would introduce a confounding variable of shear stress and would also be unable to affect Erk secretion at the basal surface of the monolayer. The results of our drug perturbation are included in Results now (lines 597+):

"Finally, we investigated the impact of modifications to cell signaling on the attention maps. Here, we perturbed the canonical MDCK model cell system with a drug selected to impact epidermal growth factor (EGF): namely, TAPI-1, which has been shown to inhibit spatial signaling and extracellular signal-regulated kinase (Erk) activation, and thereby collective migration[53,54]. The results of this experiment (see Methods) are shown in S20 Fig and indicate a striking difference relative to unperturbed tissues (e.g. Fig. 2). Specifically, EGF disruption nearly abolished the relative importance of immediate forward neighbors, shifting the focus to immediate left and right neighbors. Again, this shift would be difficult to detect by observation alone, although future work is needed to elucidate the underlying mechanism."

See also *Methods*: "*For TAPI-1 experiments, MDCK-II cells were prepared as previously described, but 2 µL of TAPI-I (Selleck) at 10mM concentration in DMSO was added to each dish.*"

Additionally, supplemental figure S20 Fig was added to display TAPI-1 experimental results on the attention plots.

The results would be improved by making a clearer distinction throughout between predictive power in the model and causative biological influence. For example, in the discussion of accuracy vs biological relevance, higher accuracy means that you have better predictive power if you know info about cells farther away from you. This is not the same as "this cell knows what's happening 3 cell lengths away from it". As the manuscript does mention higher accuracy does not always yield rules with biological relevance, it would be helpful to present (in supplements) weight maps generated from the models in Figure 2C and C' as training accuracy goes up. As accuracy goes up during training, do the patterns in attention maps become more clear, or do differences emerge during the training process?

We agree that the clarification of this point is crucial and have updated the text in a few places. For example, in:

(Lines 310+): "*It is essential to remember this key distinction as larger network structures are explored: predictive power in the model may not directly indicate causative biological influence*."

We also previously discussed this specifically in the following section (lines 403-411*):*
"The link between network accuracy and neighborhood size reflects an important and counter-intuitive design consideration since the cells we analyzed here, unlike fish, only have direct, physical awareness of their true contiguous nearest neighbors. Hence, while the accuracy increases with increasing number of nearest neighbors accounted for by the network, as more

information can be obtained over a wider spatial range, an individual cell has a more limited biological sensing regime. Thus, an increase in accuracy with increasing neighborhood size may not reflect biological realities of the system, and may instead result from the network learning more longer-range interactions. Given this, it may be helpful to configure attention networks to match the desired biological questions or constraints rather than exclusively pursuing accuracy."

Additionally, we take Reviewer 2's suggestion to display weight maps, in the supplements, generated as training accuracy goes up.

We generated the attention maps after increasing steps (in epochs) in the training process. The Results section text was modified to reflect this addition:
"*Attention maps generated after different training steps (in epochs) are shown in S5 Fig., and demonstrate convergence of the attention maps to the fully trained result; these maps correspond to the training validation accuracy plots shown in S4 Fig. With increasing accuracy, the attention maps refine to produce clearer patterns of learned relative neighbor influence by spatial location.* "

Likewise, an additional supplemental figure (S5 Fig) was added to reflect the training time reduction attention maps.

The manuscript would benefit from further exploration of the models, specifically the pi function, which should show how neighbors influence the focal cell, and the weight parameters other than xi,yi (speed, for instance). To address this, we have the following suggestions:

Include a supplementary exploration of pi as in Heras, et al. Figure 2 to show if the influences of cells in different regions differ among cell types or just the weights.
This could be used, similar to Heras, et al., to assign regions of parameter space as attraction/repulsion/alignment zones, if they exist. This would test the authors' proposal (line 422) that the MDA-MB-231 high weights represent a repulsion zone.

Previously, when this project began, we generated the attraction/repulsion/alignment zones utilizing the code from Heras et al. directly. However, one of the biggest challenges for our work in such massive cellular ensembles was generating interpretable output plots and we found the original visualizations from Heras et al. to not be well suited to our system. We attributed this to physical contact amongst cells in 2D rather than the true independent, well-separated agents in a fish school as in Heras et al.

Below, we provide some of these Heras-style plots for our standard MDCK case (10 neighbors, 20-minute prediction timestep, 10-minute time intervals). We here show the MDCK results because, in theory, these cells should be more readily interpretable than the MDA-MB-231's; however, we found even the plots for this canonical cell type to be difficult to interpret directly in a physical sense. For the same experimental conditions as our standard MDCK experiment, the attraction/repulsion/alignment/anti-alignment plots are:

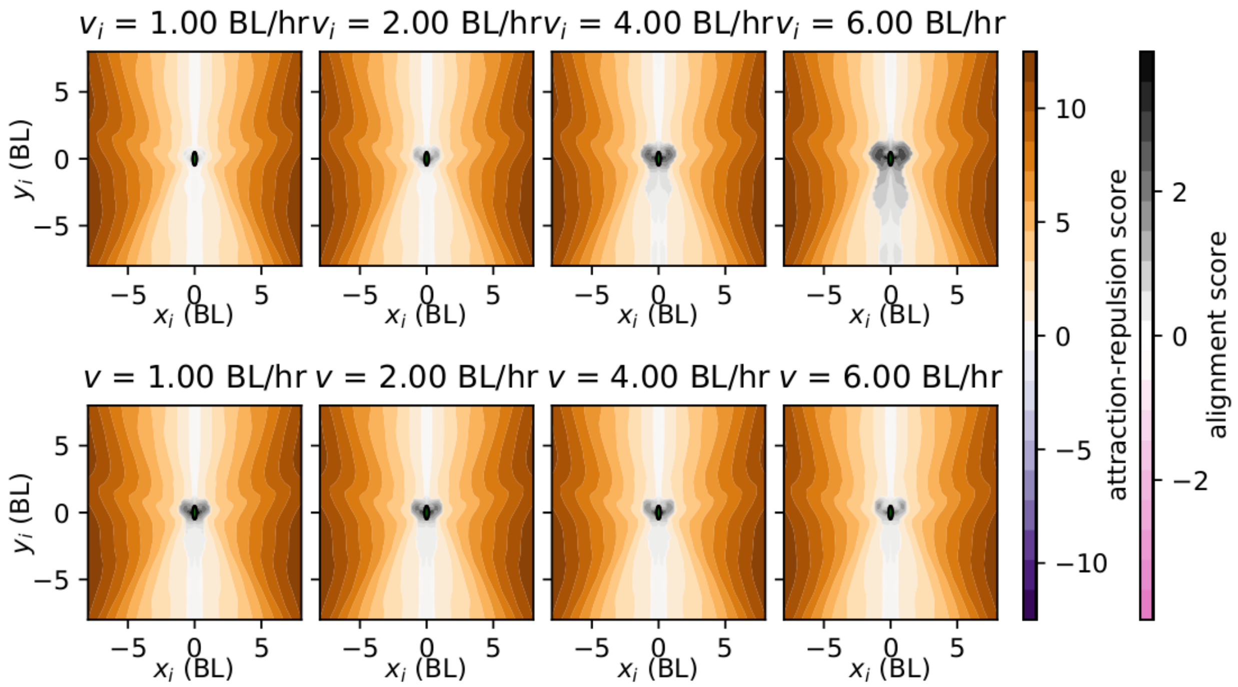

Plots here are generated using identical code to that in Heras et al. These are in contrast to the visualization strategies we developed and used instead to better emphasize the learned relative influence of neighboring cells on cellular turning dynamics in the focal cell. ***Given this discussion, we opted not to include these plots in our current work, although we note that the infrastructure to generate them is included in Heras et al. if needed by others.***

This could also help address an important question: the weight maps look highly similar for the bulk MDCK cells (described as 'trapped') and the MDA-MB cells (described as 'random'). Do the network outputs allow us to distinguish these two cases in any way?

This is also an interesting question! We thought about this and developed a new quantification of our attention map to distinguish dense or jammed cells (MDCK) from more diffusive cells (MDA-MB-231), both of which would exhibit radially symmetric attention maps. Here, we generate radial average profiles of the raw attention map data for the visually similar MDCK and MDA-MB-231 attention maps. These data are shown in S19 Fig and clearly indicate something important. MDCK cells exhibit a strikingly precise and well localized radial zone of ‘high attention’ neighbors when jammed that, critically, does not overlap with the location of the focal cell. This makes sense as it indicates a hard-core of repulsion around the focal cell as a given focal cell cannot be arbitrarily compressed. However MDA-MB-231 cells exhibit a much broader radial attention zone, indicating that neighboring cells literally crawled on top of, and across, a given focal cell, suggesting far less structured, more diffusive motion. We also note that comparisons across the respective accuracy plots for these two cell types indicate a similar story. The jammed cells exhibit largely time-step invariant accuracy, while the more random, metastatic cells actually exhibit a *decrease* in accuracy over time. These results are summarized in the text as follows in lines 581+.

“Interestingly, these data raise an important point about comparison between, and analysis of, attention maps. For instance, the attention maps of highest weighted neighbors appear visually similar at first glance between metastatic (Fig. 4D) and jammed epithelia (Fig. 5D”) despite vast differences in cell behaviors. However, quantifying these attention maps by radial averaging revealed a key difference (S19A Fig). Specifically, MDCK cells exhibited a strikingly localized radial zone of ‘high attention’ neighbors that, critically, does not overlap with the location of the focal cell. This makes sense and indicates a hard-core of repulsion around the focal cell. However, MDA-MB-231 metastatic cells exhibited a broad attention zone that overlapped with the focal cell, consistent with cells literally crawling across the focal cell and suggesting less structured motion overall. A comparison of MSD between dense epithelia and metastatic cells emphasized this lack of structure (S19B Fig.). This was further supported by comparison of the accuracy plots (Figs. 3D and 4E) that showed that MDCK prediction accuracy increased with time lags while MDA-MB-231 accuracy decreased with increasing time lags.”

Similarly, the attention maps are focused on strength of influence vs xi, yi, but W is a function of speed and acceleration as well; somewhere it would be useful to show these effects. Do some cell types pay more attention to faster cells, and others to nearer cells? What about the other inputs?

The impact of excluding certain input parameters to the model was explored in the original paper via variable blinding (the process of systematically excluding input variables to assess how the network results are affected). Additionally, we assess the impact of speed thresholding per the reviewer’s request, but found no meaningful change in the attention maps as a function of faster/slower cells. This was assessed by generating the attention maps using a speed threshold based on the median speed in the cell system.

Results section text modified to reflect this change (lines 269-271): “*Attention maps were additionally generated for slower and faster cells in the system independently (above/below a median speed threshold), but no structural difference in the plot was observed (see S6 Fig).*”

Additionally, the supplemental figure S6 Fig was added.

In the Methods, although we recognize that much of the detail can be found in Heras, et al. 2019, the authors should provide additional key information in the text. In particular, it would be better to define the network structure of the pairwise interaction function (how many layers, how many nodes on each layer, fully connected or not, etc.). Also, it would be more helpful if the authors provide more detailed information about data: exactly how many videos are used, roughly how many trajectories are in each video and of what duration, are data divided into training, validation and test groups by experiment, by tissues within the experiment, by trajectory, or by parts of trajectories?

We agree that the addition of these details is helpful and important.

The Methods section text was revised to include the following (lines 765-772): "*Each pairwise interaction block consists of a fully connected network with 3 layers of 128 neurons each followed by rectified linear unit (ReLU) operators, plus a final output layer of one neuron. These blocks are also anti-symmetrized. The weight function blocks are identical except that there is an exponential function after the final one-neuron layer, and the input is accepted in a y-reflection-invariant form. The output of the weight blocks multiply the output of the corresponding pairwise interaction blocks for each neighboring agent. All pairwise interaction blocks share the same weights.*"

Likewise, (line 728): "*MDCK and HUVEC data was collected at 10 min/frame (49/140 frames in total, respectively), while MDA-MB-231 were given 5 min/frame (97 frames total), with temporal resolution increased for the MDA-MB-231 cells to improve tracking quality.*"

And, (lines 753-758): "*In total, 13 individual tissue timelapse movies were collected for the HUVEC cell system; 15 movies for each MDCK cell system, and 17 movies for the MDA-MB-231 cell system. Independent dishes were held out from the training dataset for testing purposes. With data pre-processing, each timelapse movie for the HUVEC system resulted in approximately 70,000 data points, compared to approximately 300,000 for MDCKs and approximately 100,000 for MDA-MB-231s.*"

Figures 2 and 4 would benefit from clarification about how the attention maps and closest neighbors are normalized. From the figures, they appear to be normalized to the maximum values in the heat maps. If that is the case, it would be nice to additionally show the absolute value of these heatmaps and to see whether there are differences between different cell types.

Based on our network structure, the attention weight values only provide information pertaining to relative strength of neighbor influence. We clarified the normalization in the text within the Methods section (lines 794-796):
"*Attention weights are normalized in the range of 0-1 based on the maximum and minimum attention weight values in the test set; only relative weight strength is considered here.*"

It is not clear from the Methods, but it would be interesting to see the model trained on one experiment and tested on a separate experiment with the same cell type. Or, attention maps from replicate networks trained independently on separate experimental replicates. This may be a substantial amount of work, but is important to the claim that the network is learning cell-type-specific behaviors. If this was done, this should be clarified in the Methods.

The utilization of held-out independent tissues/movies in the test set was done and we clarified this in an earlier Methods section update for Reviewer #3.

An additional analysis that could improve the manuscript is to test the dependence of the results on the number of cells and duration of trajectories used. For example, how many cells are needed to train such a network and how does accuracy vary w/ # of training data points?

This is particularly relevant to the potential use of the model distinguishing different parts of the tissue (e.g. bulk vs edge) - how many edge cells do we need to be confident in the comparison? How will the results be affected if we have more bulk than edge cells?

We varied the number of trajectories utilized in the training set per this request, and report the accuracy results and corresponding attention maps in S18 Fig.

The Results section text was revised to reflect this additional test and to address the question (lines 543-550): *Noting that the edge regions contained ~30% fewer cells overall than the bulk, we also provide attention maps representing reduced training datasets (by including only a fixed number of trajectories) for the MDCK bulk region and edge region cases (as well as for the HUVEC cell system), allowing us to ensure a sufficient amount of data was collected (S18 Fig). The qualitative nature of the attention maps may or may not change with an increasing training set size; in general, users should assess whether or not the model itself adequately predicts the collective forward system dynamics for their use case.*

Minor:

The font size on figures is too small on axis numbers and labels, as well as legends.

We have increased the font size for axis numbers and labels as well as legends.

Why is the qualitative form of the influence map so different when the # of neighbors varies (e.g. Fig 3E*)?

We updated the notes pertaining to Fig 3E to reflect our understanding of this qualitative difference (lines 328-332): "*As the number of neighbors taken into consideration by the network increases, a wider spatial range of interactions may be considered for forward motion prediction. With an increased range from which dynamic information can be directly captured from neighboring agents, we can observe shifts in relative learned influence of neighbors; for example, as longer-range neighbors provide richer information pertaining to dynamic shifts in the forward direction than immediate forward neighbors*."

Line 410 suggests the goal of using the MDA-MB-231 cells was to test if even in apparently uncoordinated cells there is an "underlying behavioral mode"; by the end of the paragraph these cells are designated a "negative biological control". This is a subtle point, but which is it?

We agree that this is confusing language and removed the text about the "underlying behavioral mode".(line 471)

The paragraph starting at line 359 could better emphasize that the shuffling method shuffles social but not asocial data for each trajectory. This is important because it helps explain that the small increases in accuracy reflect social data alone.

We agree and have modified the text to read, "*To account for these two difficulties, we compare the standard network accuracies to accuracies derived from a network trained using shuffled trajectories: specifically, where social but not asocial data is shuffled for each trajectory. A difference in accuracy values indicates that the network captures collective phenomena.*" (line 421-424)

A histogram format for the closest neighbors plots would help with comparison to the highest weighted plots.

These plots were included as a supplemental figure (S7 Fig) and the main text was updated to reflect this in (lines 260-261): "Supplemental analogous histograms of the closest neighbor plots for all three main cell systems are provided in S7 Fig for comparison."

Typos:
Line 135: importance --> important
Line 184: additional "."
Line 256, 257: Fig S3 and Fig S5
Line 433: (C) closest neighbor scatter plot
Methods: line 591 says 3 uL and line 595 says 4 uL but they appear to be for the same experiment
Line 644: "[9]"
Line 688: "]\"
Line 650-651: Reference not in the reference format (Heras et al.)
Line 660: "TrackMate plugin *in* ImageJ"

We have made all these changes in the text (lines numbers will be different due to other edits).
