# Supplementary material for: Learning the rules of collective cell migration using deep attention networks
Source: PLoS Comput Biol. 2022 Apr 27;18(4):e1009293. doi: 10.1371/journal.pcbi.1009293 (PMC9106212; doi:10.1371/journal.pcbi.1009293)
Supplement: Attachment — Submitted filename: FinalRevResponse_AttentionNetwork.pdf [file pcbi.1009293.s026.pdf]

Dear Colleagues,

Our final revision responses are included below, in-line and in blue.

Thank you for your consideration,

Prof. Daniel J. Cohen and the crew.

Reviewer's Responses to Questions

Comments to the Authors:
Please note here if the review is uploaded as an attachment.

Reviewer #2: The authors have addressed all my comments, including the control study on the Viscek model, which has improved the paper. I therefore recommend publication. I would like to congratulate the authors on a great manuscript that I am confident will be of interest to a broad audience.

> *Response: We appreciate the feedback as well as the suggestion to include a Viscek model as that was a compelling demonstration to validate our network.*

Reviewer #3: We appreciate the authors have put in a considerable amount of effort to improve the clarity and scientific rigor of their manuscript, and think some of the new results are very exciting. We recommend publication after the following minor points are addressed.

1. The results concerning the impact of extracellular signaling on model assumptions are very exciting (Figure S20). However, these findings really demonstrate the power of the model and are potentially under-discussed in the text. For example, the finding that the focus shifts to left and right neighbors from the forward neighbors could be placed in more of a biological context even if the mechanism is unclear.

> *Response: We were also excited by these results, but we prefer to be cautious when it comes to interpreting machine learning results without underlying biological mechanisms. However, in response to the reviewer's excitement we have modified our textual presentation of the results in S20. Here, we have created an additional section header calling out detection of external perturbations, and added additional text to the discussion and interpretation. Additionally, we have added a stronger emphasis in the discussion section of the importance of future studies exploring attention networks to complement biological mechanistic studies. See lines 597-614 and 648-651.*
>
> *"Finally, we investigated the impact of modifications to cell signaling on the attention maps. Here, we perturbed the canonical MDCK model cell system with a drug selected to*

impact epidermal growth factor (EGF)--TAPI-1--which has been shown to inhibit spatial signaling and extracellular signal-regulated kinase (Erk) activation, and thereby collective migration[52,53]. The results of this experiment (see Methods) are shown in S20 Fig and indicate a striking difference relative to unperturbed tissues (e.g. Fig. 2). Specifically, EGF disruption nearly abolished the relative importance of immediate forward neighbors, shifting the focus to immediate left and right neighbors. This shift in relative attention away from the forward neighbor and towards the lateral neighbors likely reflects the network detecting underlying biomechanical differences induced by EGFR/Erk signaling disruption as prior molecular studies have connected MDCK front-rear polarity to EGFR/Erk signaling[54]. While future work may be needed needed to verify and elucidate the specific molecular mechanisms, there are two key points to emphasize. First, this resulting shift in attention is not easily apparent from visual observation alone, emphasizing the importance of attention works for detecting subtle, collective responses to perturbations. Second, the attention network detected and clearly highlighted a connection between Erk and neighbor coordination without any foreknowledge of biased assumptions from the user, which makes it a powerful tool for hypothesis generation and screening of complex cellular dynamics datasets.

"Attention mapping may eventually help to connect biophysical mechanisms to collective behavior 'rules', as is hinted at in the ability of the network to detect how chemical disruption of EGFR/Erk signaling reprograms collective attention (S20 Fig.)."

2. The section "Biophysical and biological variations affect the attention maps" may be more accurately renamed "Biophysical, biochemical, and biological variations affect the attention maps" with the addition of the results described in Figure S20.
Response: We agree with the sentiment and created an entirely separate section header to better emphasize the biochemical perturbation detection (see above). The new title is "Detection of collective behavior changes in response to external perturbations"

3. We appreciate the authors for clarifying how the attention weights are normalized. It would be helpful to understand why they only consider relative strength important and if the absolutely strengths from different cell lines are comparable or not.

Response: This is an important question that deserves a separate follow-up study. In our present study, the network learns the dynamics of each cell line individually, and the network is structured such that the learned attention weights are normalized at training-time, to act as multipliers to the output of the cell-line-specific interaction-network modules. So as a result, there are no truly meaningful absolute values which would allow cross-cell-line comparison at the level of the weights. However, the attention maps themselves are directly comparable. Future work may improve the normalization or provide experimental conditions that would make more direct comparison of the weights themselves more informative.

4. The new Figure S18 clearly illustrates in some cell types there are significant changes in the qualitative nature of the attention maps. It would be helpful if the authors expanded on why in some cases varying the trajectory number has such a large impact.

Response: Depending on the cell type, it may be that by forcing the network to predict cell dynamics over long trajectories, the network learns to improve prediction accuracy by analyzing a wider range of neighbors within specific domains. For example, for a cell line with high persistence, it may be advantageous to analyze cells well behind the focal cell, in an effort to anticipate compression fronts which may propagate to impact the motion of the focal cell after a number of trajectory steps into the future. This is why it is crucial to consider the information a focal cell is likely to have access to biologically: how might these dynamics differ if the cell can only take information directly from its immediate neighbors? The use of the attention maps allows us to probe individual cell lines and vary trajectory lengths, attention radii, and other relevant input variables.

5. Line 512-513 should cite the results demonstrating that accounting for larger numbers of nearest neighbors does not obviously impact the network accuracy results.

Response: We have now updated the lines in question to point to S12F Fig. where the accuracy vs. neighbor # and time step is presented for MDA-MB-231

6. The Figure 3 legend says to see Figure S6 for a matching study in HUVEC cells, but the text and supplemental figures suggest this should be referencing Figure S9.

Response: We have made this correction, thank you for noticing.
